# Electromagnetic drag forces between HTS magnet and tube infrastructure for hyperloop

**DOI:** 10.1038/s41598-023-39916-7

**Published:** 2023-08-03

**Authors:** Suyong Choi, Minki Cho, Jungyoul Lim

**Affiliations:** 1 New Transportation Innovative Research Center, Korea Railroad Research Institute, Uiwang, Korea; 2 Korea National University of Transportation, Uiwang, Korea

**Keywords:** Electrical and electronic engineering, Mechanical engineering

## Abstract

Maglevs are typically accelerated using electromagnetic propulsion and levitation. High-temperature superconducting (HTS) magnets along with electrodynamic suspension (EDS) and linear synchronous motors are one of the best options for Hyperloop. However, the strong magnetic fields generated by HTS magnets on the pods inevitably interact with the magnetic and conductive structures in the vacuum tubes, along with the tube itself, while the pods move through the tubes. This interaction is observed as a drag force on the pods, significantly reducing the propulsion efficiency. This study comprehensively analyzes the electromagnetic drag force (EDF) generated by HTS magnets on pods, which accounts for most of the drag forces faced by Hyperloop. Theoretical analysis and 3D FEA simulations are performed to analyze the propulsion forces with HTS magnets and all the drag forces on the pods. The EDF generated by AISI 1010 steel rebars in concrete guideways is even greater than the designed propulsion forces of 40 kN. Consequently, high-manganese (Hi-Mn) steel and insulated steel rebars are adopted and analyzed using 3D FEA simulations. The EDFs generated by the AISI 1010 steel and Hi-Mn steel vacuum tubes are determined by varying the distance between the HTS magnets and tubes at 50 and 1200 km/h, respectively; a minimum distance of 0.75 m is determined by a drag force below 8 kN within their operating velocities. Lastly, the total EDFs of the AISI 1010 steel and Hi-Mn steel tubes with EDS rails are obtained through the optimal design of rebars and tubes. The simulation results show that the total EDFs can be significantly reduced to below 10 kN (approximately 25% of the designed propulsion force after the levitation of pods).

## Introduction

Hyperloop, which presents a maximum velocity of 1200 km/h in near-vacuum tubes of 0.001 atm, has recently gained considerable interest in the development of future transportation systems^1–6^. Hyperloop pods move through near-vacuum tubes to significantly reduce the air drag, which is one of the major factors affecting the velocity of ground transportation systems. Typically, electromagnetic propulsion and levitation systems (EPLSs) are used to accelerate the pods to their maximum velocity. Steel tubes^7,8^ are considered the best material for the construction of near-vacuum tubes due to their high vacuum tightness. At the same time, a research is being conducted on high-density concrete tubes^9^, which present a lower construction cost than steel tubes. Combinations of various EPLSs have been analyzed for the EPLSs of Hyperloop. For example, electrodynamic suspensions (EDSs) using permanent magnets and electromagnetic suspensions (EMSs) using electro-magnets have been widely implemented in levitation systems, and most Hyperloop companies have shifted from the EDS levitation system to the EMS system^7,8,10,11^ due to its simplified system configuration. However, the EMS presents a higher infrastructure construction cost owing to the small air gap between the pods and levitation coils. Conversely, linear induction motors (LIMs), which comprise conductive plates on the pods and electromagnets on the tubes, and linear synchronous motors (LSMs), which comprise permanent magnets on the pods and electromagnets on the tubes, are primarily used for the propulsion system of Hyperloop. LIM systems are relatively simple and easy to build; however, LSMs exhibit better propulsion performance than LIMs at high velocities^12^. This is the main reason behind the recent shift in the propulsion type by most Hyperloop companies from the LIM to the LSM^7,8,10,11^ with permanent magnets. A research team led by the Korea Railroad Research Institute (KRRI) has made considerable progress in the Hyperloop project since 2008^12–20^. They have been developing the high-temperature superconducting (HTS) magnets^14^ on pods for propulsion and levitation systems; by applying a thermal battery instead of an on-board cooling system, the temperature rise of the on-board HTS magnets can be significantly suppressed, therefore, the on-board HTS magnets are free from the cryogenic cooling system and power supply system, which can minimize their power consumption and weight on pods^15^. For the rapid and accurate analysis of the EDS, several researches have been conducted using equivalent inductance models^16^ and the simplified levitation-coil model of the EDS^17^. In general, the propulsion forces of LSMs are directly proportional to the magnetic fields generated by the magnets on the pods^18^, and the levitation forces of the EDSs are proportional to the square of the magnetic field generated by the magnets on the pods^19^. Thus, EPLSs, with HTS magnets generating strong magnetic fields, produce larger air gaps than other EPLSs, which can reduce the construction cost and spatial safety margin^20^ while the pods move at their maximum velocity. The strong magnetic fields generated by the HTS magnets inevitably interact with all the magnetic and conductive structures in the tubes, as well as the tube itself. This interaction is observed as a nonnegligible electromagnetic drag force (EDF) on the pods^21–23^, which significantly reduces its propulsion efficiency and accounts for most of the total drag force on the pods of Hyperloop. So far, to analytically calculate the EDF and create its general formulation, several studies have been conducted^24–26^; however, the suggested general formulations for the EDF^24,25^ are only available with a single magnet generating uniform magnetic fields, when the air-gap between magnets and materials is zero and skin-depth does not exist. Moreover, the EDF using AC circuits^26^ was introduced to obtain a general form, however, this model is only available for fitting curves in the post-process phase after simulation results are obtained.

This paper presents a comprehensive analysis of the EDF generated by HTS magnets on pods, which accounts for most of the drag forces in Hyperloop. First, the configuration of the EPLS with HTS magnets developed by the KRRI and the system design parameters are explained in detail. Subsequently, all the drag forces on the pods are evaluated using theoretical and simulation analyses. The results demonstrate that the mechanical and aerodynamic drag forces are negligible, and the optimum blockage ratio (BR) is 0.6 at a vacuum tube pressure of 0.001 atm. The proposed design approaches for rebars and vacuum tubes are optimized in terms of their fabrication method and the distance between the HTS magnets and tubes by using high-manganese (Hi-Mn) steels and AISI 1010 steels. The results are verified using 3D FEA simulations. Finally, this paper presents a full-sized 3D model, including steel tubes and EDS rails, and the total EDFs are obtained within its operating velocities.

## Methodology

### Overall configuration of the EPLS with the HTS magnet

**Figure 1:**
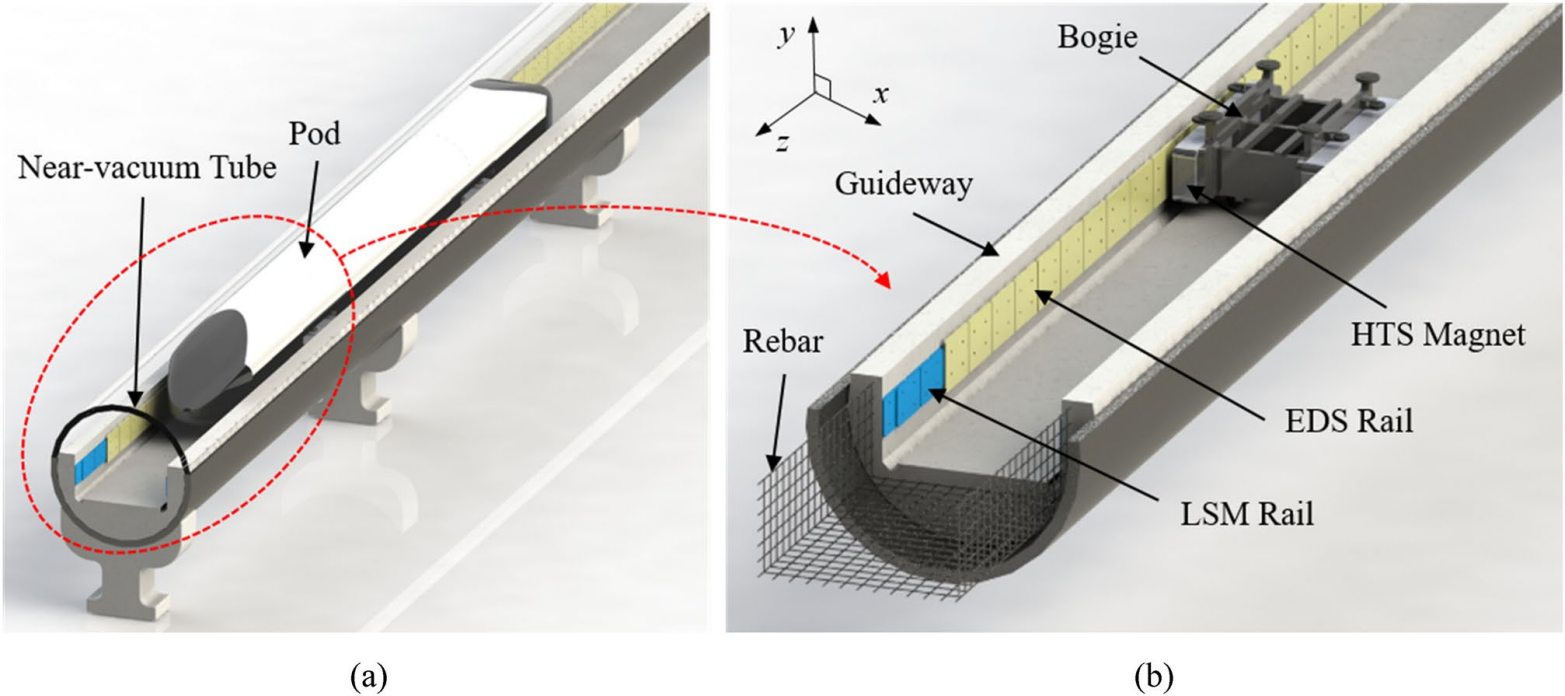
Overall configuration of Hyperloop. (**a**) Bird’s eye view. (**b**) Cross-sectional view.

As shown in Fig. 1, Hyperloop includes two subsystems: (1) the pod subsystem comprising pod bodies; HTS magnets; bogies; and heating, ventilation, and air conditioning (HVAC), and (2) the vacuum-tube subsystem, which includes guideways, vacuum pumps, an LSM, and EDS rails. The pods are moved through a near-vacuum tube with a pressure of 0.001 atm, which is generated by vacuum pumps in the tube, to avoid air drag forces at high velocities. To ensure a vacuum-tight assembly, the tubes must be fabricated using ferromagnetic materials, such as steel, non-magnetic materials such as Hi-Mn, and non-conductive materials such as high-density concrete. The pods in the tube move along the concrete guideways and reinforced rebars are installed in the guideways to endure that three-dimensional forces are generated by the magnetic interaction of the HTS magnets with EPLSs, that is, the LSM and EDS rails.

The LSM and EDS rails overlap in the guideway and are installed on both the walls facing each HTS magnet. With HTS magnets, propulsion and levitation can be achieved simultaneously. In the bogie, HTS magnets, including multi-magnetic poles, are symmetrically installed on both sides, and the pods levitate over a take-off velocity, ${v}_{l}$, of 150 km/h with an air gap of 50 mm between the HTS magnets and guideways.

When the pods move through the tube, the strong magnetic fields generated by the HTS magnets pass through all the conductive and ferromagnetic structures in the tube as well as through the tubes themselves, and the total EDFs, *F*_*d*_, are likely to significantly affect the pods. If the rebars and tubes are fabricated using non-conductive and non-magnetic materials, such as fiber-reinforced plastic (FRP), they are free from EDFs. However, their construction cost, construction time, and mass manufacture must be considered for the commercialization of Hyperloop.

In addition, in the passenger cabin, magnetic fields generated by HTS magnets should be mitigated under the electromagnetic field regulation, e.g., ICNIRP guideline 2009^27^; 0.4 T for the general public and 0.5mT for the people with implanted electronic medical devices.

**Figure 2:**
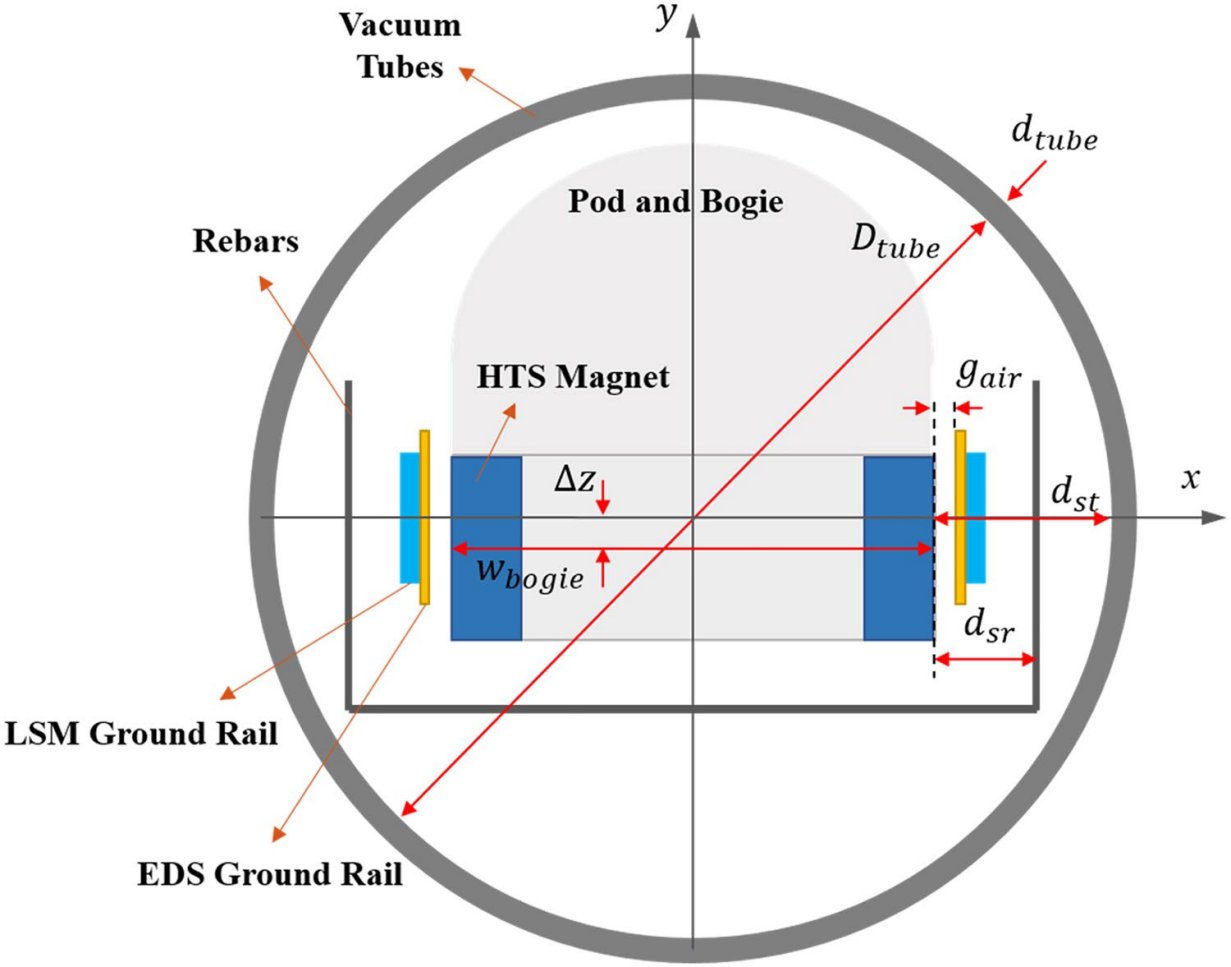
Cross-sectional view and parameters of Hyperloop for EDF analysis.

For the EDF analysis, the overall configuration of the Hyperloop system is simplified using the design parameters of conductive and ferromagnetic structures, as shown in Fig. 2. First, the rebars in the guideways are located inside the tube, where *D*_*tube*_ and *d*_*tube*_ represent the diameter and thickness, respectively. Additionally, the vertical center of the LSM and EDS rails is aligned with that of the HTS magnets at Δ*z* of zero before ${v}_{l}$. Subsequently, the pods levitate with an air gap, *g*_*air*_, between the EDS rails and HTS magnets, and this levitation leads to an offset Δ*z* of 0.05 m. *d*_*sr*_ represents the distance between the surface of the HTS magnets and the center of rebars, and *d*_*st*_ represents the distance between the surface of the HTS magnets and the inside of the tubes.

The length, *L*_*pod*_, and mass, *m*_*pod*_, of the pods are determined by considering the maximum passenger capacity, and the cross-sectional area of the pod, *A*_*pod*_, is then obtained. *N*_*p*_ denotes the total number of HTS magnetic poles, which are evenly distributed on both sides of the bogie. The pods move on their own mechanical wheels until *v*_*l*_ and then start to levitate by a maximum driving velocity, *v*_*m*_, of 1200 km/h, as shown in Table 1.

**Table 1:** Given requirements and nominal parameters of Hyperloop.

Parameter	Value	Unit
Total mass of Hyperloop pod, ${m}_{p}$	$\mathrm{20,000}$	$\mathrm{kg}$
Length of pod, ${L}_{pod}$	20	$\mathrm{m}$
Cross sectional area of pod, ${A}_{pod}$	$4.91$	${\mathrm{m}}^{2}$
Passenger seats	$20$	–
Maximum acceleration, ${a}_{p}$	$2.0$	$\mathrm{m}/{\mathrm{s}}^{2}$
Diameter(thickness) of tube, ${D}_{tube}({d}_{tube})$	3.6 (0.1)	$\mathrm{m}$
Total number of HTS magnet poles, ${N}_{p}$	12	–
Air gap, ${g}_{air}$	$0.05$	$\mathrm{m}$
Width of bogie, ${w}_{bogie}$	$2.1$	$\mathrm{m}$
Distance between HTS magnet and rebar, ${d}_{sr}$	$0.27$	$\mathrm{m}$
Distance between HTS magnet and tube, ${d}_{st}$	$0.75$	$\mathrm{m}$
Maximum driving velocity, ${v}_{m}$	333.33 (1200)	m/s (km/h)
Take off velocity, ${v}_{l}$	41.67 (150)	m/s (km/h)

**Figure 3:**
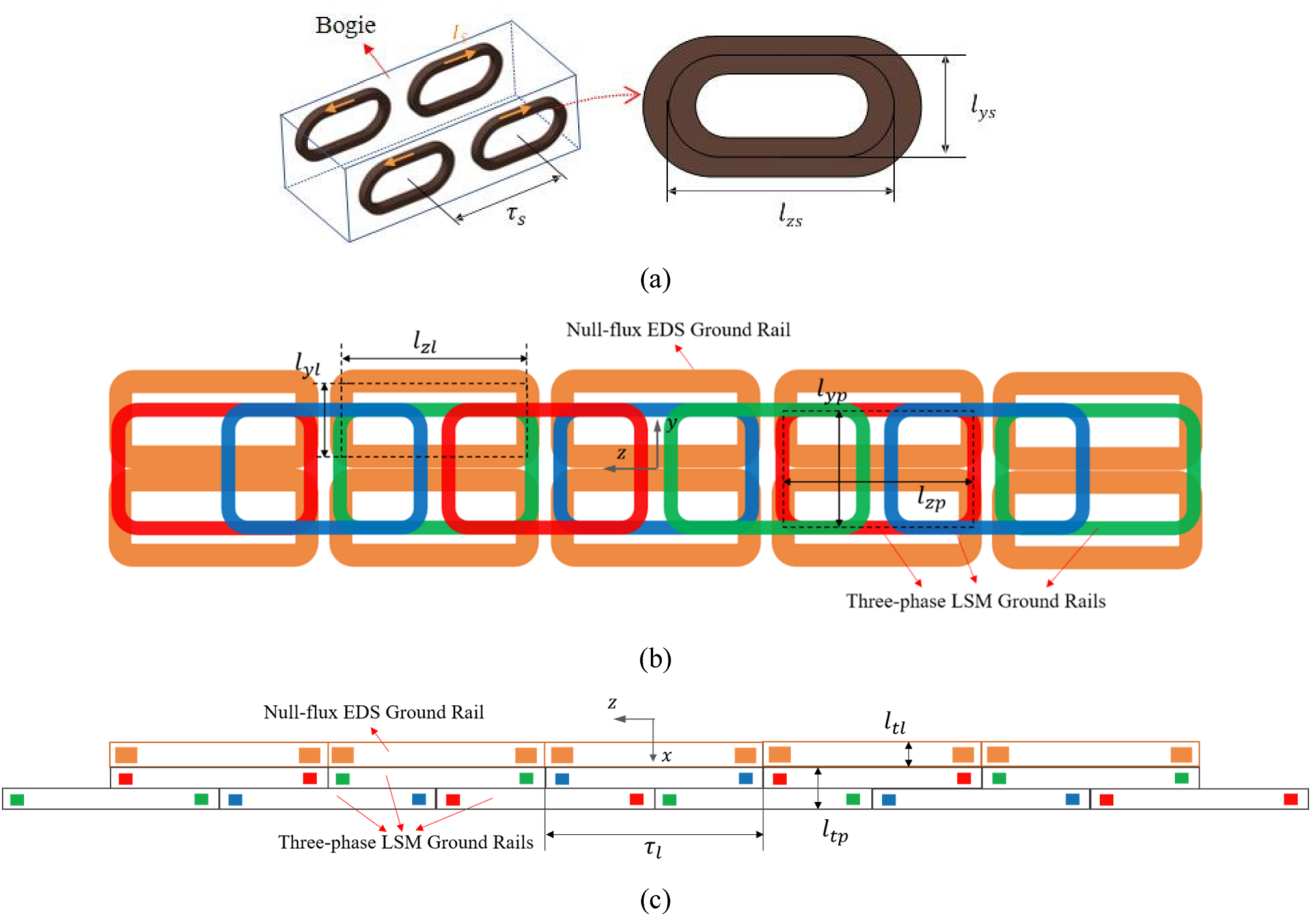
Configuration of the proposed LSM and EDS rails with HTS magnet. (**a**) Simplified bogie with 4 pole–2 module HTS magnets. (**b**) Side view and (**c**) top view of one-sided LSM and EDS ground rails.

An electromagnetic drag analysis of Hyperloop is then performed, where a bogie includes a 4 pole–2 module HTS magnet, as shown in Fig. 3a. Additionally, double-layer, three-phase, and concentrated winding-type LSM ground rails are adopted for efficient propulsion, as shown in Fig. 3b and c. EDS ground rails are installed on the LSM ground rails and connected using the null-flux method to mitigate the EDF. Table 2 lists the design parameters of the HTS magnets, LSM, and EDS ground rails.

**Table 2:** Parameters of HTS magnets, LSMs, and EDS ground rails for electromagnetic drag analysis.

Parameter		Value	Unit
HTS magnets	Magnetomotive force, ${\mathcal{F}}_{s}$	$600$	${\mathrm{kA}}_{\mathrm{DC}}\cdot$Turns
	Operating current, ${I}_{s}$ Number of HTS magnet turns, ${N}_{s}$	$150$ 4000	$\mathrm{A}$ Turns
	Pole pitch, ${\tau }_{s}$	$1.2$	$\mathrm{m}$
	Effective coil sizes, ${l}_{zs}\times {l}_{ys}$	$0.9\times 0.4$	${\mathrm{m}}^{2}$
LSM/EDS ground rails	Ground rail pitch, ${\tau }_{l}$	$0.8$	$\mathrm{m}$
	Effective LSM rail sizes, ${l}_{zp}\times {l}_{yp}$	$0.64\times 0.48$	${\mathrm{m}}^{2}$
	Number of LSM rail turns per phase, ${N}_{l}$	5	$\mathrm{Turns}$
	Cross sectional area of LSM rail	120	${\mathrm{mm}}^{2}$
	Effective EDS rail sizes, ${l}_{zl}\times {l}_{yl}$	$0.69\times 0.22$	${\mathrm{m}}^{2}$
	Number of EDS rail turns, ${N}_{e}$	16	Turns
	Cross sectional area of EDS rail	100	${\mathrm{mm}}^{2}$
	Thickness of LSM and EDS tracks, $({l}_{tp}, {l}_{tl})$	$(0.03, 0.05)$	$\mathrm{m}$
	LSM phase current, ${I}_{l}$	$1000$	${\mathrm{A}}_{\mathrm{rms}}$

Prior to full-scale prototype HTS magnets of 600 kAT introduced in the Table 2, a couple of the small-scale REBCO magnets with HTS wires of SuNam^28^ were designed and fabricated to evaluate its electrical and mechanical performance. One of the HTS magnets was introduced^15^, and the parameters of HTS magnets, i.e., the number of turns and critical current, were determined using the Victoria University of Wellington's superconducting wire database^29^. In fact, SuperPower wires were used for the real-scale HTS magnets of 700 kAT^30^ and it was found that the operating current was set to 150 A, which is lower than the performance of the HTS wires, because the safety margin of 25% was applied to the operating current at the operating temperature of 40 K.

In addition, the AC loss^31^ and mechanical strength of the HTS magnets are also important factors to be considered for the real-scale HTS magnets. In order to withstand the 3-axes forces generated on the HTS magnets, supporters made of glass fiber reinforced plastics (GFRPs) and pipes made of stainless steels were generally used. For the real-scale HTS magnets, increasing the diameter as well as number of the supporters and pipes would be primarily considered to withstand the forces mentioned in this manuscript, however, this approach also lead to increasing the conduction heat load into the HTS magnet, therefore, the optimal design process, e.g., mass of a solid nitrogen (SN_2_), should be conducted for the thermal battery. For the evaluation of AC losses on the HTS magnet, this topic will be discussed in future studies.

### Propulsion force analysis with HTS magnets

When the power angle, $\delta_{p}$, is 90°, under *l*_*zs*_ = *l*_*zp*_ and *l*_*ys*_ = *l*_*yp*_ , the maximum propulsion force, *F*_*p*_, of the Hyperloop pod can be simplified as follows^18^:

1 $$ F_{P} = c_{o} N_{s} I_{s} N_{l} I_{l} \sin \delta_{P} , $$

where $c_{0}$ denotes the design constant factor, which is proportional to the number of inverter phases and LSM rail sizes. It is inversely proportional to the pole pitch of the HTS magnet, $\tau_{s}$, and exponentially decreases with an increase in the air gap, $g_{air}$. $I_{s}$ and $I_{l}$ denote the phase currents of the HTS magnets and LSM rails, respectively. $N_{s}$ and $N_{l}$ represent the number of turns per phase for the LSM rails and HTS magnets, respectively.

**Figure 4:**
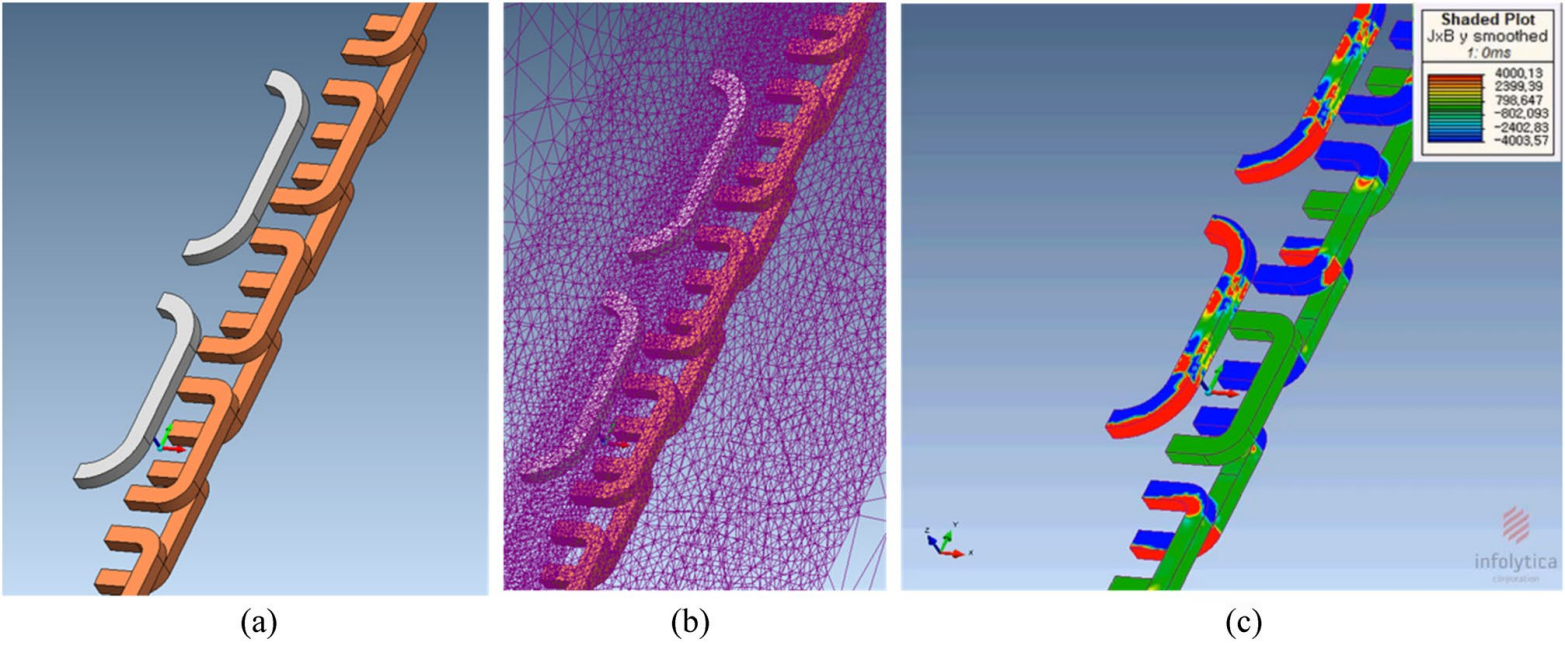
3D half-models (Siemens MagNet)^32^ of the propulsion force, *F*_*p*_, with simplified 2 pole–1 module HTS magnet. (**a**) Bird’s eye view. (**b**) Bird’s eye view with meshes. (**c**) Shaded plot of J $\times$ B on *y*-axis.

Because the *F*_*p*_ is not numerically calculable using the given geometries and parameters, the FEA simulation was used to determine the main design parameters of LSM rails such as $I_{l}$, $N_{l}$, $l_{zp}$, and $l_{yp}$, as shown in Fig. 4. To obtain the constant propulsion force, the required phase current,$I_{l}$, of the LSM rails is fixed when the other parameters are time-invariant. However, the required phase voltage, $V_{T}$, of the LSM rails increases with an increase in the velocity of the pods, resulting in an increase in $E_{l}$ and $\omega_{s}$. In the frequency domain, the phase voltage, ${\mathbf{V}}_{T}$, of the LSM rails for each phase can be summarized as follows:

2 $$ {\mathbf{V}}_{T} = {\mathbf{I}}_{l} (R_{l} + j\omega_{s} L_{l} ) + {\mathbf{E}}_{l} , $$

where $R_{l}$ and $L_{l}$ represent the total resistance and inductance of the LSM rail for each phase, respectively. ${\mathbf{E}}_{l}$ denotes the voltage induced by the LSM rail in each phase. $\omega_{s}$ denotes the angular frequency used for current control, which is proportional to the velocity of the pods.

Additionally, with an increase in velocity, the required phase voltage $V_{T}$ increases when $I_{l}$ is fixed. This leads to an increase in the apparent power, $S$, for the LSM rails, which can excessively increase the capacity of inverters for the LSM rails, particularly at a maximum velocity of 1200 km/h.

3 $$ S = 3V_{T} I_{l} , $$

*S* denotes the complex power, which is equal to the maximum capacity of the LSM rails and the minimum capacity of the inverters.

Therefore, to maximize the use of the inverter capacity, the pods are accelerated with constant propulsion forces of up to 600 km/h. From 600 km/h to 1200 km, the pods accelerate by reducing the propulsion forces to maintain constant power, which is the maximum inverter capacity. This inverter control can only be achieved without any drag forces on the pods, whereas the acceleration of the pods with drag forces is inversely proportional to the total drag forces, $F_{td}$, which can be summarized as follows:

4 $$ a_{p} = \frac{{F_{p} - F_{td} }}{{m_{p} }}, $$

where $a_{p}$ is the acceleration of the pods, $F_{p}$ is the effective propulsion force, and $m_{p}$ is the mass of the Hyperloop pod. $F_{td}$, can be determined by summing the aerodynamic drag forces (ADFs), $F_{a}$, mechanical friction forces of the rolling tires, $F_{f}$, and total EDFs, $F_{d}$, as follows:

5 $$ F_{td} = F_{a} + F_{f} + F_{d} , $$

The following sections present a detailed explanation of each drag force based on its theories and simulations. The detailed profiles of $F_{p}$ and $I_{l}$, along with the operating velocities for inverter control, are introduced at the end of the Section “Electromagnetic drag forces by steel tubes and EDS rails”.

### Aerodynamic drag forces inside the vacuum tubes

Several design parameters affect the aerodynamic drag forces (ADFs), *F*_*a*_, of the pods in the vacuum tubes, which include the length, forehead, cross-sectional shape of the pods, pod velocity, pressure and temperature in tubes, and BR. Here, BR is the ratio of the cross-sectional area of an empty vacuum tube, *A*_*tube*_, to that of the pod, *A*_*pod*_; it can be expressed as follows:

6 $$ {\text{Blockage}}\;{\text{ratio}}\;\left( {{\text{BR}}} \right) = \frac{{A_{pod} }}{{A_{tube} }}. $$

The ADFs are directly proportional to the air density in the tube, and they exhibit a nonlinear relationship with the length and forehead of the pods, their velocity, and BR^33^. However, the analysis of ADFs without a high BR design is insufficient for commercializing the design of Hyperloop. This is mainly because the construction cost of the infrastructure of vacuum tubes, which accounts for a third of its total construction cost, is strongly dependent on the diameter of the tube, *D*_*tube*_, which has not been considered in previous studies.

**Figure 5:**
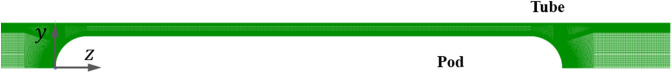
2D half-model (ANSYS Fluent) of aerodynamic drag forces corresponding to its pod velocities at a BR of 0.6.

Determining the minimum diameter of tubes within the appropriate ADFs for the proposed Hyperloop system is essential. Therefore, ADFs with different BR values and various velocities are obtained in this study based on computational fluid dynamics (CFD) by using a commercial software (ANSYS Fluent), where the length of the pods is 20 m, and the temperature and pressure in the tubes are 300 K and 0.001 atm, respectively, as shown in Fig. 5.

**Figure 6:**
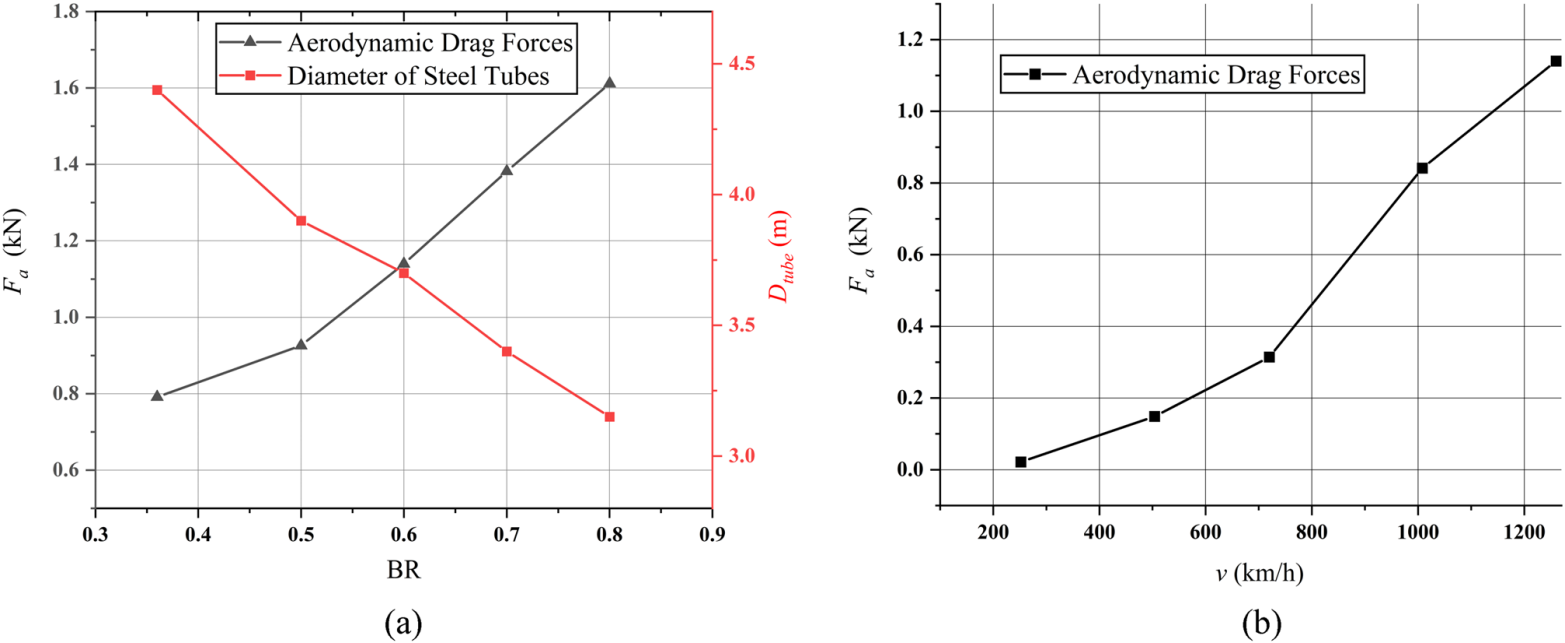
CFD simulation results of aerodynamic drag force (ADF) based on its velocity and BR. (**a**) ADF and *D*_*tube*_ corresponding to BRs at a velocity of 1260 km/h. (**b**) ADF corresponding to its velocities at a BR of 0.6.

Typically, the ADFs are proportional to the BRs, whereas *D*_*tube*_ is inversely proportional to the BRs, as shown in Fig. 6a. For example, with a BR of 0.6, the ADFs are negligible when compared to the designed propulsion forces of 40 kN within its operating velocities at a pressure of 0.001 atm, as shown in Fig. 6b. However, when the pressure within the tubes increases by 0.01 atm at the same BR, the ADFs significantly increase by 11.8 kN and are no longer negligible. *D*_*tube*_ must be less than 4 m considering the manufacturing cost of steel tubes for Hyperloop. This is because steelmaking companies such as POSCO are already producing steel plates and tubes for other purposes, which means that no additional facilities are required to manufacture tubes for Hyperloop. Therefore, a higher BR is better for commercialization in terms of the infrastructure cost of Hyperloop.

In summary, with a BR of 0.8, the maximum ADF of 1.6 kN is much smaller than the designed propulsion force of 40 kN at the maximum operating velocity, as shown in Fig. 6a. This indicates that a BR of 0.8 is one of the options considering the infrastructure cost for Hyperloop. However, for now, the minimum *D*_*tube*_ is determined not by the ADFs but by the EDFs generated by the HTS magnets in the tubes. This is because the EDFs are generally much larger than the ADFs, and the EDFs are inversely proportional to the *D*_*tube*_, which will be discussed in the Section “Electromagnetic drag forces by steel tubes”. In addition, as shown in Fig. 19, the optimal distance between HTS magnet and tube, *d*_*st*_ of 0.75 m, is determined by the allowable drag force and the *d*_*st*_ leads to the optimal BR of 0.6.

### Mechanical friction forces of rolling tires

For Hyperloop adopting the EDS levitation, the pods move along the guideways using their own wheels before *v*_*l*_, and then start to magnetically levitate at the maximum driving velocity, *v*_*m*_, without any mechanical contact. Thus, the mechanical friction forces (MFFs) of the rolling tires, *F*_*f*_, must be considered before *v*_*l*_. They can be calculated as follows:

7 $$ F_{f} = c_{rr} m_{pod} g, $$

where *m*_*pod*_ is the mass of the pod, *c*_*rr*_ is the rolling resistance coefficient, and *g* is the gravitational force of the Earth.

When the pods with car tires move on asphalt guideways, a *c*_*rr*_ of 0.015^34^ can be used similar to conventional vehicles. An *F*_*f*_ of 3 kN is obtained using Eq. (7); this mechanical friction force is higher than the maximum ADFs of 2 kN at a velocity of 1260 km/h with a BR of 0.8. However, the impact of *F*_*f*_ is only valid until the *v*_*l*_ of 150 km/h; therefore, the impact is limited within the overall velocities.

### Electrodynamic drag forces of HTS magnets

The strong moving magnetic fields generated by the HTS magnets on the pods pass through all the conductive and ferromagnetic structures in the tube, as well as the tubes themselves. Therefore, the total EDFs, *F*_*d*_, which are likely to occur significantly on the pods, can be summarized as follows:

8 $$ F_{d} = F_{dl} + F_{dg} + F_{dt} , $$

where *F*_*dl*_ is the force originating from the EDS coils, *F*_*dg*_ is that originating from the rebar, and *F*_*dt*_ is that originating from the tube. In the following section, each EDF is analyzed using 3D FEA simulations, and several technical approaches are introduced to mitigate *F*_*d*_.

## Results

When the pods move, the strong magnetic fields generated by the HTS magnets on the pods induce currents on all the conductive structures in the tubes as well as the conductive tubes themselves. The induced currents, which include eddy currents, *I*_*e*_, and loop currents, *I*_*lp*_, form opposite magnetic poles on the conductive structures and tubes and these currents interact with the HTS magnets. This magnetic interaction is observed as EDFs on the pods in accordance with Faraday's law and Lenz’s law and is given as follows:

9 $$ e = - \frac{{d\phi_{m} }}{dt}, $$

where *e* is the electromotive force, and $\phi_{m}$ represents the magnetic fluxes passing through materials.

**Figure 7:**
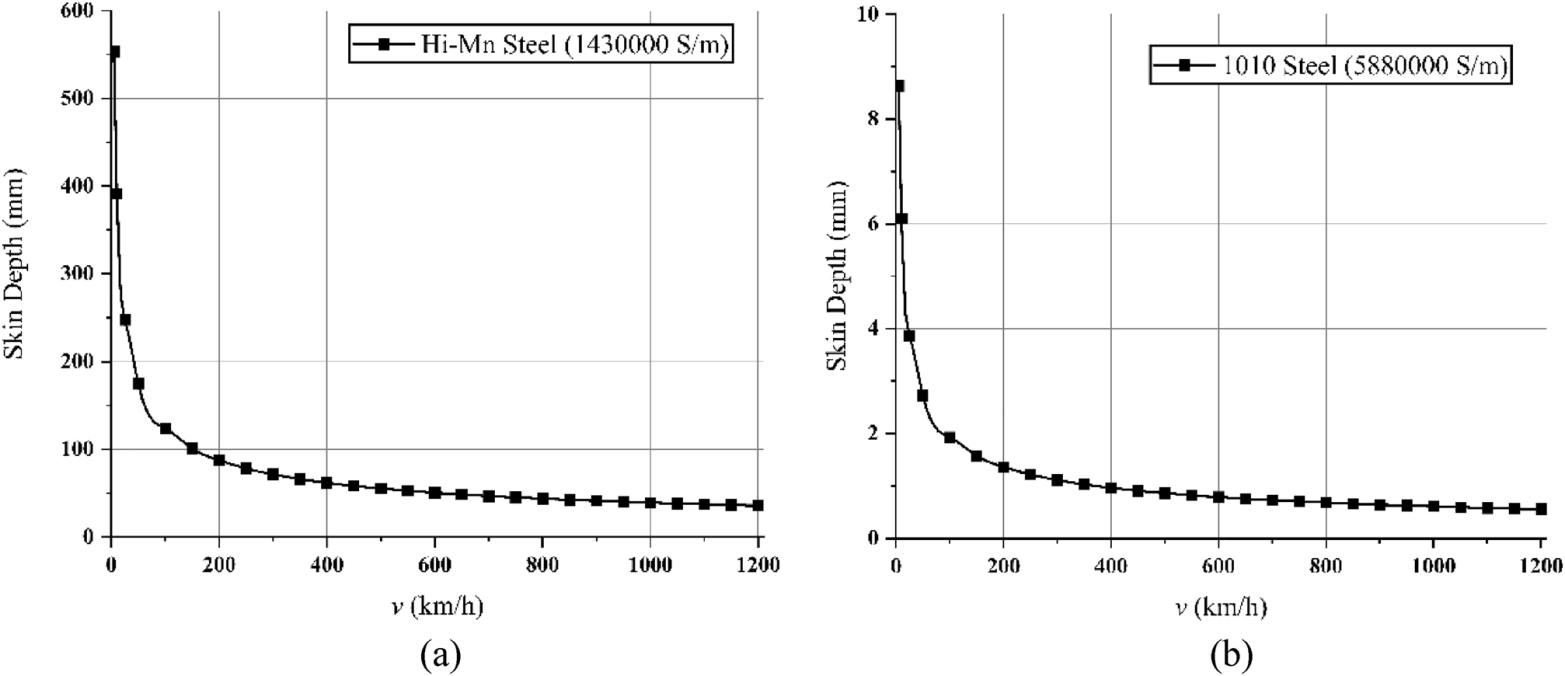
Calculation of the skin depth for (**a**) Hi-Mn steels having ${\mu }_{r}$ of 1 and (**b**) 1010 steels having ${\mu }_{r}$ of 1,000 at 20 ℃.

In addition, the AC resistance of rebars and tubes could be highly affected by skin effects, which make induced currents mainly flow into the surface of materials at high frequencies and significantly increases the AC resistance, as shown in Fig. 7. Owing to skin effects, the skin depth^35^ can be expressed as follows:

10-1 $$ \delta = \frac{1}{{\sqrt {\pi f_{s} \mu_{0} \mu_{r} \sigma } }}, $$

where $\delta$ is the skin depth (m), $f_{s}$ is the operating frequency (Hz), $\mu_{0}$ is the magnetic permeability of free space, $\mu_{r}$ is the relative permeability, and $\sigma$ is the electrical conductivity (S/m). Here, $f_{s}$ generated by the moving HTS magnets^18^, which is the operating frequency of induced currents on the surface of materials, can be obtained as follows:

10-2 $$ 2\tau_{s} f_{s} = v \to f_{s} = \frac{v}{{2\tau_{s} }}. $$

**Figure 8:**
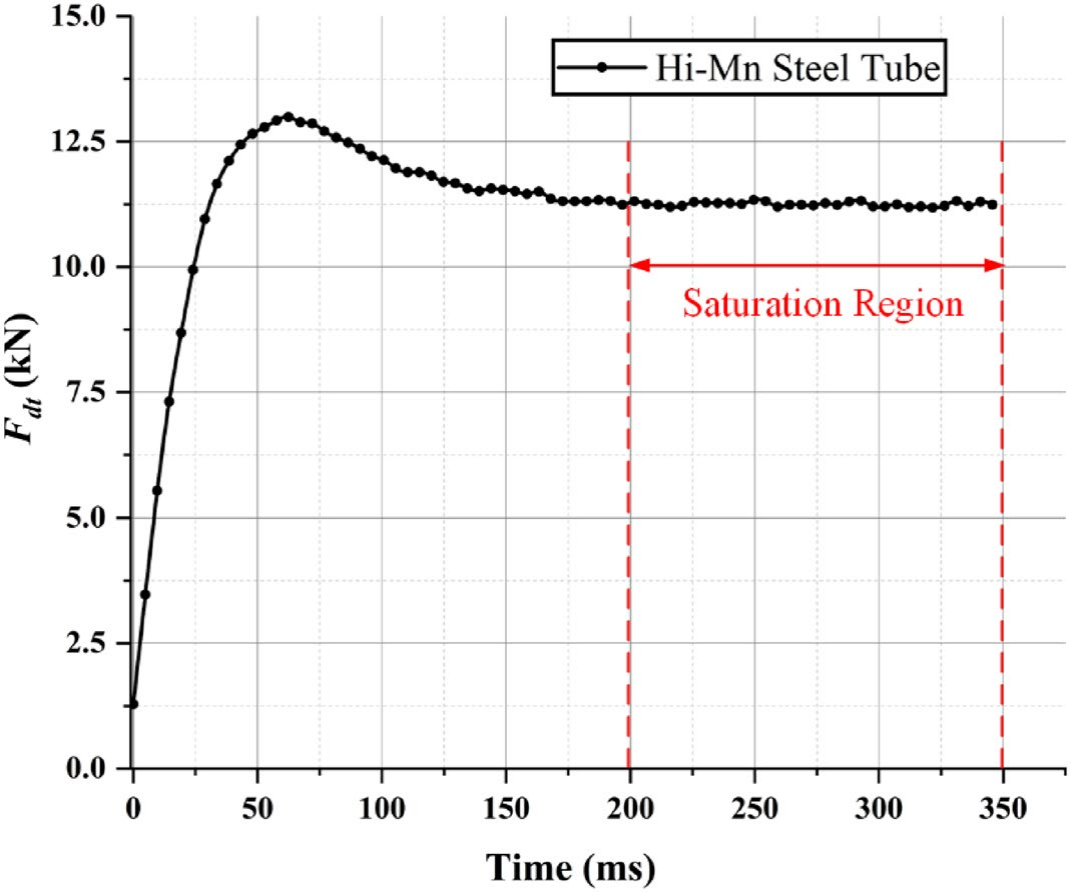
Electromagnetic drag force—time variation for the Hi-Mn steel tube using a 12 pole-6 module HTS magnet. From 200 ms, the drag forces start to saturate. The total simulation time is 350 ms with *v* of 50 km/h.

As mentioned in the introduction section, the EDF is not numerically calculable using a general formulation because magnetic fluxes generated by HTS magnets and induced currents from (9) flow into complicated magnetic and electrical paths, respectively. In addition, the ferromagnetic material, i.e., AISI 1010 steel, and skin-depth could be the main sources that increase the complexity of the magnetic and electrical paths. Therefore, the FEA simulations are the only way to accurately determine the EDFs with HTS magnets on the pods. Therefore, by using Hi-Mn and AISI 1010 steels, three different models were constructed with rebars, tubes, and EDS rails, and 3D FEA simulations (Siemens MagNet) were conducted to obtain each EDF. In addition, as shown in Fig. 8, it was found that the electromagnetic drag force starts to saturate after 200 ms for the Hi-Mn steel tube using a 12 pole-6 module HTS magnet with *v* of 50 km/h at *d*_*st*_ of 0.75 m. Therefore, all the drag forces introduced in this manuscript were obtained by the saturation region. Each time step and total simulation time are determined by the unit distance step of 6 cm and given moving length of 5 m for each operating velocity, respectively. Moreover, polynomial order of 2 and conjugate gradient tolerance of 10^–6^ were used in the 3D FEA simulations.

### Electromagnetic drag forces by rebars

**Figure 9:**
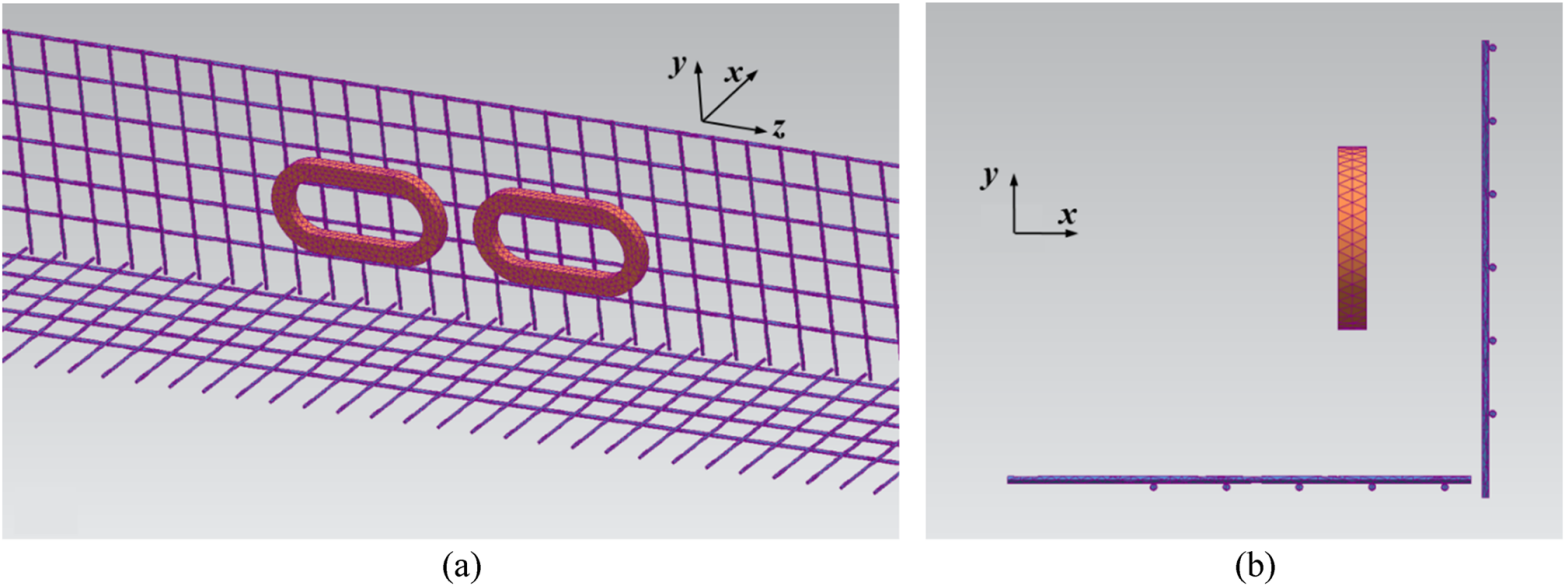
3D half-models (Siemens MagNet) of EDFs made by rebars *F*_*dg*_ with simplified 2 pole–1 module HTS magnet. (**a**) Bird’s eye view. (**b**) Front view.

A 3D half-model comprising a 2 pole–1 module HTS magnet with steel rebars was adopted to minimize the simulation time, as shown in Fig. 9a and b. This simplified approach is applicable only when the number of magnetic poles in the HTS magnets is directly proportional to the EDFs. Moreover, the B-H curve of the AISI 1010 steel is used in the simulation, as shown in Fig. 9.

**Figure 10:**
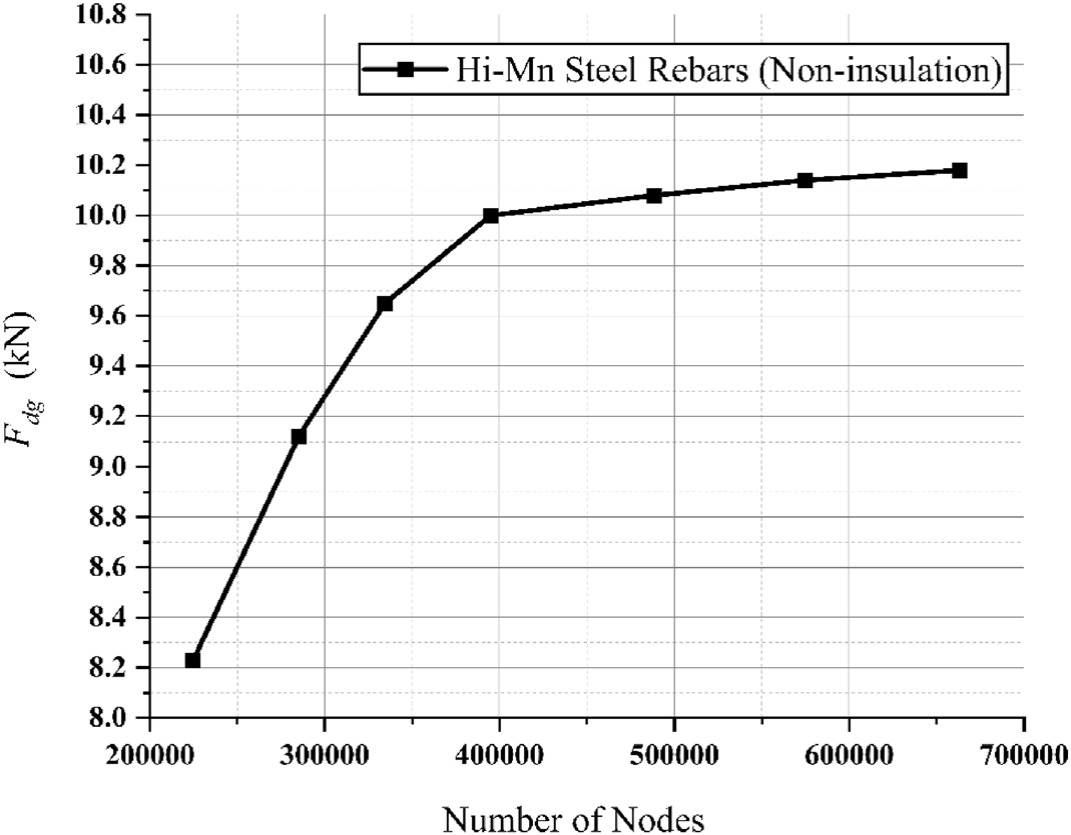
3D FEA simulation result of the EDFs produced by non-insulated Hi-Mn steel rebars, *F*_*dg*_, corresponding to various number of nodes at velocities of 200 km/h for full-scale Hyperloop including a 12 pole–6 module HTS magnet.

As shown in Fig. 10, the h-convergence was confirmed by *F*_*dg*_ corresponding to various number of nodes at velocities of 200 km/h for full-scale Hyperloop including a 12 pole–6 module HTS magnet.

In addition, a grid independence test of the mesh is conducted using the 3D FEA model for non-insulated Hi-Mn steel rebars. In the Table 3, the parameters are listed, and different meshes, i.e., coarse (488,210 nodes), medium (574,785 nodes), and fine (662,897 nodes), are compared with respect to *F*_*dg*_. The difference between coarse and fine meshes is 0.98%, while that between medium and fine meshes is 0.39%. Therefore, the medium mesh is adopted to all the steel rebar models.

**Table 3:** Parameters and results of grid sensitivity test for the steel rebar models with the conjugate gradient tolerance of 10^–6^.

Parameters	Coarse	Medium	Fine
No. of nodes	488,210	574,785	662,897
No. of tetrahedra	2,823,239	3,311,151	3,801,716
No. of model volumes	129	129	129
No. of model surface	552	552	552
Drag force (kN)	10.08	10.14	10.18
Difference (%)	0.98	0.39	0

**Figure 11:**
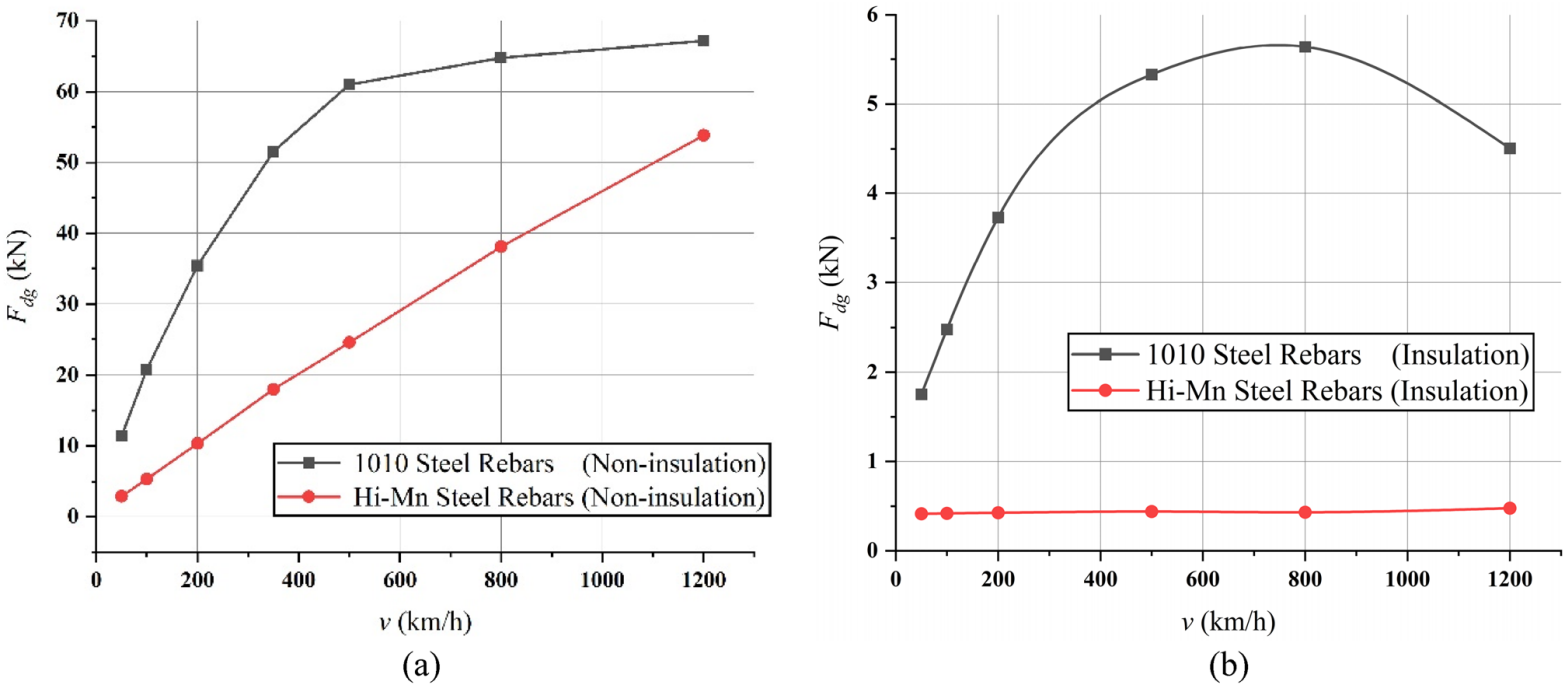
3D FEA simulation results of the EDFs, *F*_*dg*_, generated by rebars for full-scale Hyperloop including a 12 pole–6 module HTS magnet. (**a**) *F*_*dg*_ generated by Hi-Mn and AISI 1010 steel rebars corresponding to its various operating velocities for the non-insulation and (**b**) insulation cases.

AISI 1010 steel rebars are widely used for concrete guideways owing to their cost-effectiveness. However, there are two significant problems associated with using AISI 1010 steel rebars for Hyperloop. The first problem is that the *F*_*dg*_ generated by the AISI 1010 steel rebars could be even more than the designed propulsion force of 40 kN over the velocity of 250 km/h, as shown in Fig. 11a.

**Figure 12:**
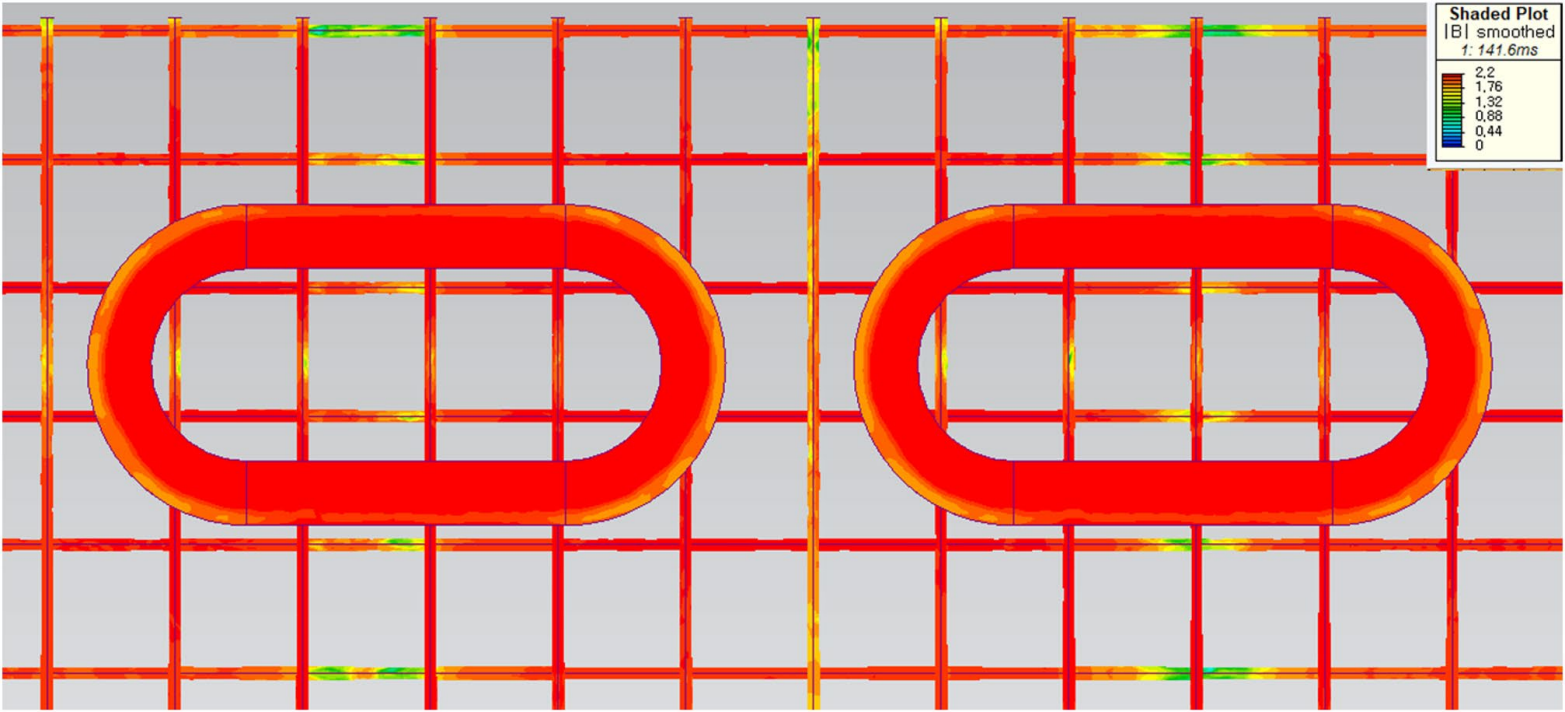
3D FEA simulation result of the 3D half-model for shaded *B* for the AISI 1010 steel tube using a 2 pole-1 module HTS magnet with *v* of 50 km/h at *d*_*st*_ of 0.75 m.

In addition, the main factor, that the *F*_*dg*_ generated by the AISI 1010 steel rebars is generally higher than that generated by the Hi-Mn steel rebars, is the hysteresis loss^36,37^ because rebars are magnetically saturated by HTS magnets as shown in Fig. 12.

**Figure 13:**
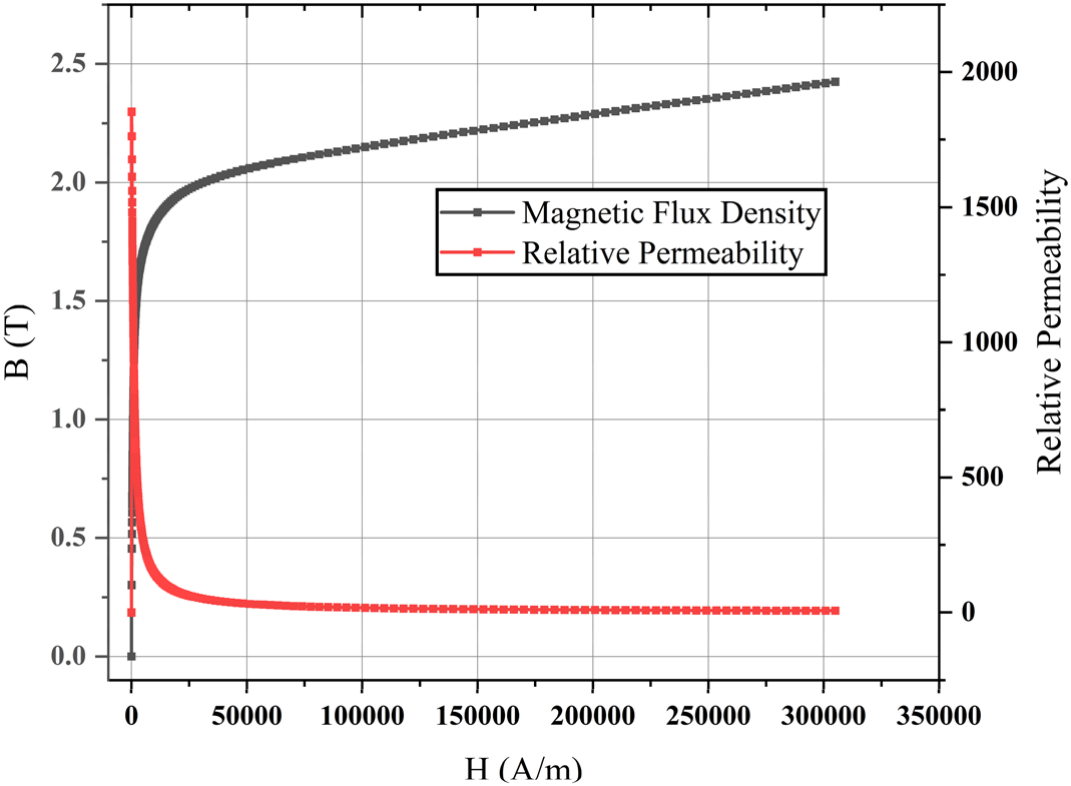
B-H curve and relative permeability of AISI 1010 steels at 20 ℃ used in the FEA simulation.

In accordance with the Fig. 13, the hysteresis loss is directly proportional to its velocity and caused in the ferromagnetic materials, i.e., the AISI 1010 steel as follows:

11 $$ P_{k} = \sigma V_{rebar} B^{n}_{\max } f_{s} , $$

where $\sigma$ and $V_{rebar}$ represent the steinmetz’s coefficient and volume of the materials, respectively. $B^{n}_{\max }$ denotes the maximum flux density in the material. $f_{s}$ denotes the frequency for the change of magnetic poles. Here, $\sigma$ and *n* depend on the material.

The other problem is that the AISI 1010 steel rebars are rapidly magnetized by the strong magnetic fields generated by the HTS magnets, and the attraction forces, *F*_*a*_, between the steel rebars and HTS magnet generate opposite forces of guidance (*x*-axis) and levitation (*y*-axis), which are the main forces contributing to the degradation of the dynamic stability of the pods. In the stationary mode, i.e., *v* is zero, the attraction force, *F*_*ag*_, which acts in the opposite direction of the guidance forces, is approximately 5.9 kN and the attraction force, *F*_*al*_, which acts in the opposite direction of the levitation forces is approximately 0.6 kN with 12 pole–6 module HTS magnets. However, when *v* increases, *F*_*ag*_ is inversely proportional to the square of operating velocities whereas *F*_*al*_ is directly proportional to the square of operating velocities in steel tubes. This effect is strongly related to the dynamics and stability of pods; therefore, so this topic will be discussed in another paper.

The second problem caused by the attraction forces can be mitigated by using Hi-Mn steels, which are not magnetized at all owing to their relative permeability of 1, when *v* is zero, and its parameters are listed in Table 4. However, the *F*_*dg*_ of Hi-Mn steel rebars is also approximately 54 kN at a maximum velocity of 1200 km/h, which is still exceedingly high to be used as rebars.

**Table 4:** Parameters of steel rebars for 3D FEA simulations.

Parameter		Value	Unit
Diameter of rebars, *D*_*rebar*_		0.020	m
Length of rebars, *L*_*rebar*_		1.275	m
Distance between rebars, *l*_*lp*_		0.200	m
Relative permeability, ${\mu }_{r}$	AISI 1010 steel	B-H curve	–
	Hi-Mn steel	1	–
Resistivity, $\rho$	AISI 1010 steel	1.7 $\times$ 10^–7^	Ω m
	Hi-Mn steel	7.0 $\times$ 10^–7^	Ω m

In detail, *F*_*dg*_ includes three different forces, that is, drag forces generated by induced eddy currents, *F*_*dg_ec*_, magnetic hysteresis, *F*_*dg_hy*_, and induced loop currents, *F*_*dg_lp*_ as follows:

12 $$ F_{dg} = F_{dg\_ec} + F_{dg\_hy} + F_{dg\_lp} . $$

**Figure 14:**
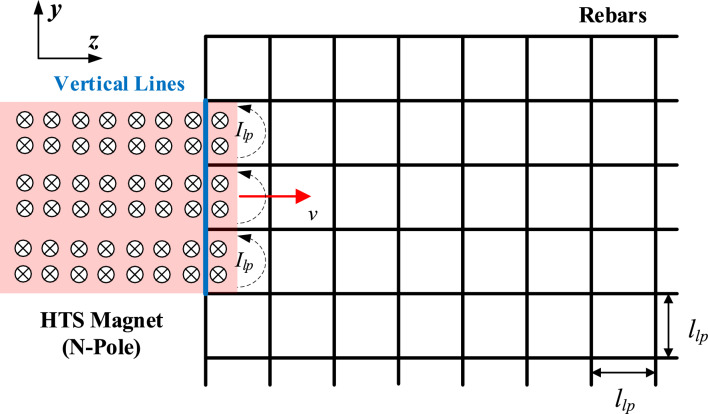
Simplified configuration of the induced loop currents created by moving HTS magnet.

Considering *F*_*dg_lp*_, the mechanical connection of conductive rebars in concrete guideways creates an electrical connection as a short circuit where induced loop currents can flow, as shown in Fig. 14.

Assuming uniform magnetic flux density perpendicularly passing through rebars, *B*_*lp*_, and constant pod velocity, *v*, the electromotive force in the single loop of rebars, $e_{lp}$, can be simplified as follows:

13 $$ \left| {e_{lp} } \right| = \left| {\frac{{d\phi_{lp} }}{dt}} \right| = \left| {(\mathrm{v} \times \mathrm{B}_{{\mathrm{lp}}} ) \cdot \mathrm{l}_{{\mathrm{lp}}} } \right| = vB_{lp} l_{lp} . $$

The induced loop current in the single loop of rebars, *I*_*lp*_, is simplified as follows:

14 $$ \left| {I_{lp} } \right| = \left| {\frac{{e_{lp} }}{{\sqrt {R_{lp}^{2} + (\omega_{s} L_{lp} )^{2} } }}} \right| = \frac{{vB_{lp} l_{lp} }}{{\sqrt {R_{lp}^{2} + (\omega_{s} L_{lp} )^{2} } }}, $$

where the resistance and inductance of the single loop of rebars are *R*_*lp*_ and *L*_*lp*_, respectively.

In accordance with the Lorentz force, assuming a straight stationary rebar, the loop drag forces, *F*_*lp*_, which only exist on the vertical line (*y*-axis) of rebars, are opposite to *F*_*p*_ and are determined as follows:

15 $$ \left| {\mathrm{F}_{lp} } \right| = \left| {I_{lp} (\mathrm{l}_{lp} \times \mathrm{B}_{lp} )} \right| = I_{lp} l_{lp} B_{lp} = \frac{{B_{lp}^{2} l_{lp}^{2} v}}{{\sqrt {R_{lp}^{2} + (\omega_{s} L_{lp} )^{2} } }} \cong \frac{{B_{lp}^{2} l_{lp}^{2} v}}{{R_{lp} }},\;{\text{when}}\;R_{lp} > > \omega_{s} L_{lp} . $$

For example, as shown in Fig. 14, each loop generates *F*_*lp*_ and *F*_*dg_lp*_ can be simply obtained by the summation of the each *F*_*lp*_ in the loop of rebars as follows:

16 $$ \left| {F_{dg\_lp} } \right| \cong \left| {3F_{lp} } \right| = \frac{{3B_{lp}^{2} l_{lp}^{2} v}}{{R_{lp} }}. $$

**Figure 15:**
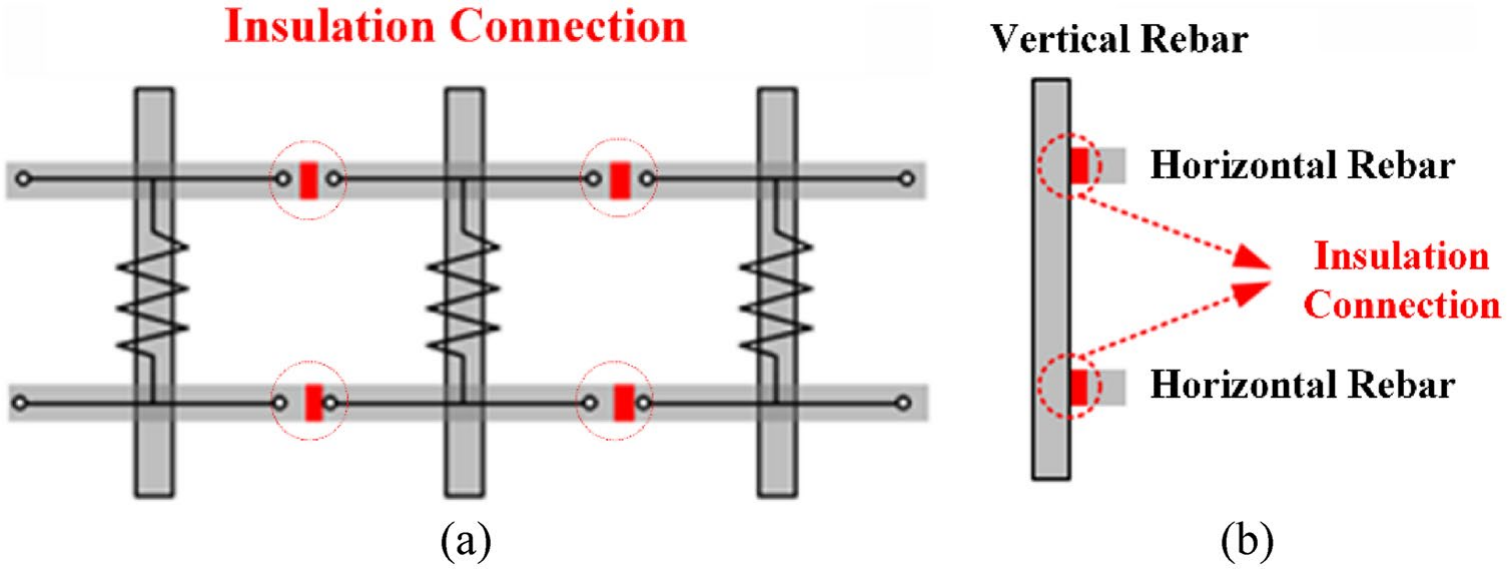
Examples of eliminating loop currents on rebars with insulation. (**a**) Front view. (**b**) Side view.

Equations (15) and (16) clearly indicate that the only way to effectively eliminate the *F*_*lp*_ as well as *F*_*dg_lp*_ is to create an electrical connection of conductive rebars in a concrete guideway as an open circuit by the electrical insulation of the rebars, as shown in Fig. 15.

For example, an insulation connection can be achieved by inserting non-conductive materials, such as FRPs and double-sided insulation tapes, on the joint in contact with the horizontal–vertical rebars and the horizontal–horizontal rebars, as shown in Fig. 15. Furthermore, an electrical open circuit, whose resistance is observed to be infinite and whose induced loop currents are actively reduced, can be achieved by using insulation layers for the joint. Therefore, *F*_*dg_lp*_ can be simplified as follows:

17 $$ F_{dg\_lp} \cong 0\;{\text{when}}\;R_{lp} = \infty . $$

**Figure 16:**
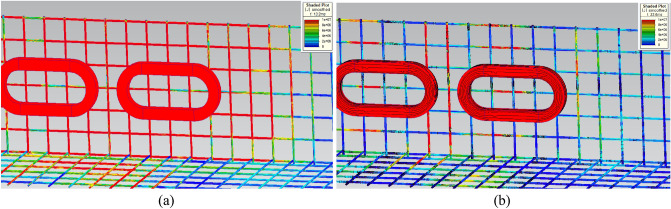
3D FEA simulation results of the current densities, *J*, generated by AISI 1010 rebars for a 2 pole-1 module HTS magnet with *v* of 200 km/h at *d*_*sr*_ of 0.27 m. (**a**) Non-insulation and (**b**) insulation rebars with the same upper bound of shaded *J* plot.

Insulated steel rebar techniques were applied to the 3D FEA simulation models to verify the insulation effect by separating horizontal rebars from vertical rebars with the air-gap of 10 mm. The simulation results demonstrated that the *F*_*dg*_ produced by the insulated Hi-Mn steel rebars decreased by approximately one-hundredth at a maximum velocity whereas the *F*_*dg*_ produced by the insulated AISI 1010 steel rebars reduced by approximately one-tenth compared with the *F*_*dg*_ produced by the non-insulated steel rebars, as shown in Fig. 11b. Furthermore, the effect of the insulation connection on rebars is clearly indicated by the 3D FEA simulation results of the current densities, *J*, as shown in Fig. 16. From these results, the *F*_*dg*_ is simplified as follows:

18 $$ F_{dg} = F_{dg\_ec} + F_{dg\_hy} + F_{dg\_lp} \cong F_{dg\_lp} . $$

Consequently, by adopting insulated Hi-Mn steel rebars, *F*_*ag*_ and *F*_*al*_ are zero at zero velocity, and an *F*_*dg*_ of approximately 0.5 kN could be considered negligible within its various operating velocities. Therefore, the total electromagnetic forces, *F*_*d*_, of Eq. (8) can be expressed as follows:

19 $$ F_{d} = F_{dl} + F_{dg} + F_{dt} \cong F_{dl} + F_{dt} . $$

### Electromagnetic drag forces by steel tubes

**Figure 17:**
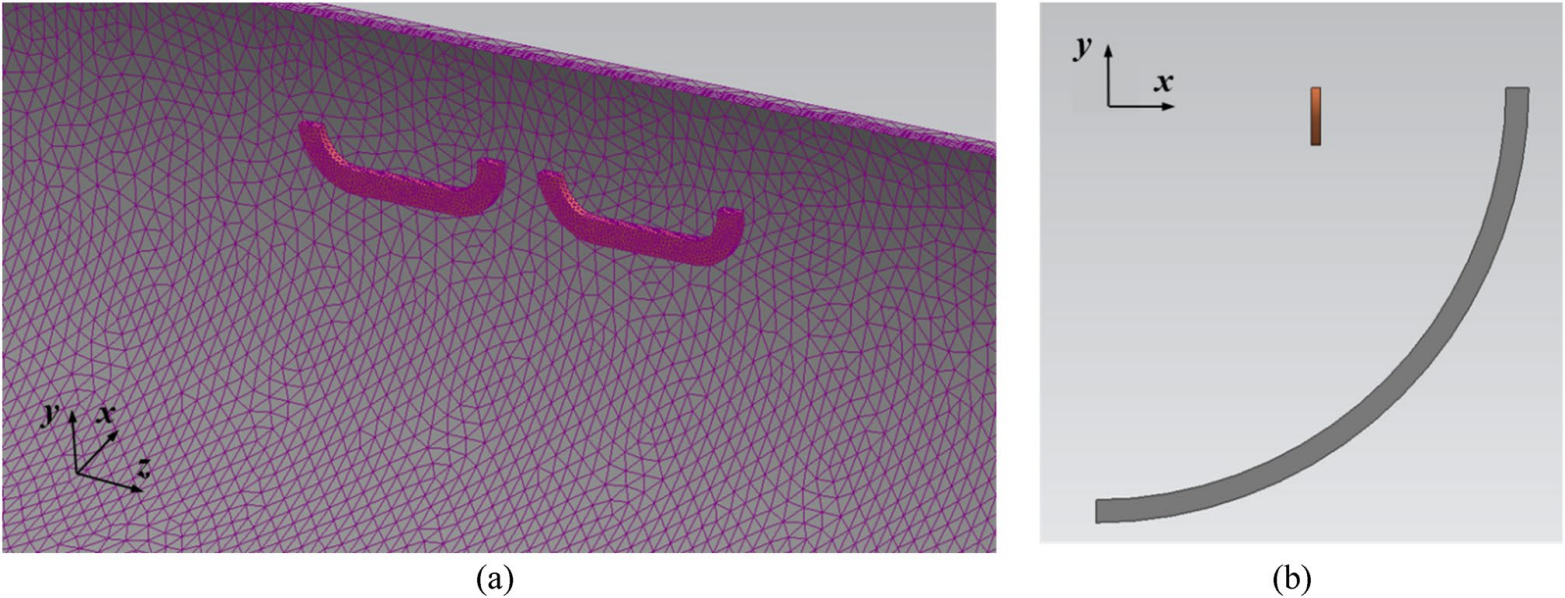
3D quarter-models (Siemens MagNet) of EDFs produced by steel tubes, *F*_*dt*_, with the half of a 2 pole–1 module HTS magnet. (**a**) Bird’s eye view. (**b**) Front view.

From Eq. (19), it can be observed that only two magnetic drag forces need to be considered: *F*_*dt*_ and *F*_*dl*_. This section presents a detailed analysis of *F*_*dt*_ produced by the AISI 1010 steel and Hi-Mn steel tubes. A 3D quarter-model, which includes the half of a 2 pole–1 module HTS magnet with steel tubes, was used to simplify the FEA simulation, as shown in Fig. 17.

**Figure 18:**
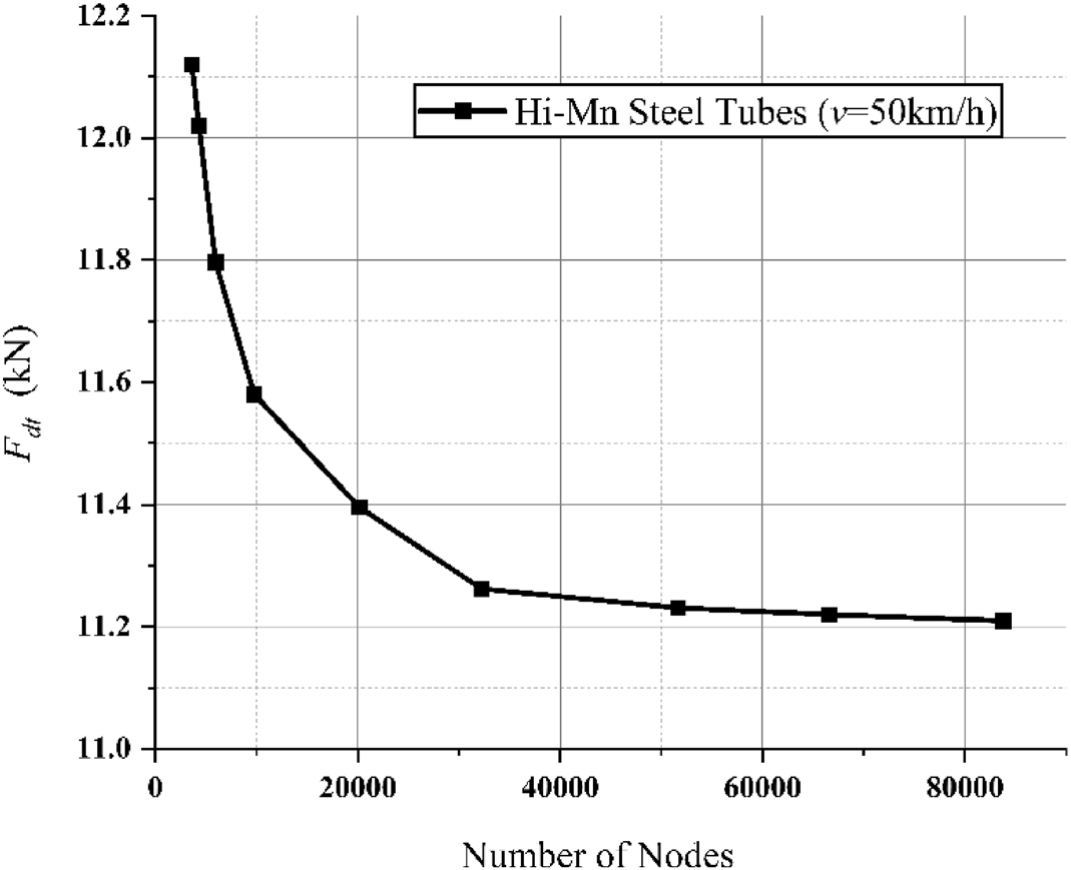
3D FEA simulation result of the EDFs produced by Hi-Mn steel tubes, *F*_*dt*_, corresponding to various number of nodes at velocities of 50 km/h for full-scale Hyperloop including a 12 pole–6 module HTS magnet.

As shown in Fig. 18, the h-convergence was confirmed by *F*_*dt*_ corresponding to various number of nodes at velocities of 50 km/h for full-scale Hyperloop including a 12 pole–6 module HTS magnet.

In addition, a grid independence test of the mesh is conducted using the 3D FEA model for the Hi-Mn steel tubes. In the Table 5, the parameters are summarized, and three different meshes, i.e., coarse (51,656 nodes), medium (83,769 nodes), and fine (112,371 nodes), are compared in terms of *F*_*dt*_. The difference between coarse and fine meshes is 0.97%, while that between medium and fine meshes is 0.43%. Therefore, the medium mesh is adopted to all the steel tube models.

**Table 5:** Parameters and results of grid sensitivity test for the steel tube models with the conjugate gradient tolerance of 10^–6^.

Parameters	Coarse	Medium	Fine
No. of nodes	51,656	83,769	112,371
No. of tetrahedra	286,935	465,144	679,054
No. of model volumes	12	12	12
No. of model surface	79	79	79
Drag force (kN)	11.256	11.196	11.148
Difference (%)	0.97	0.43	0

**Figure 19:**
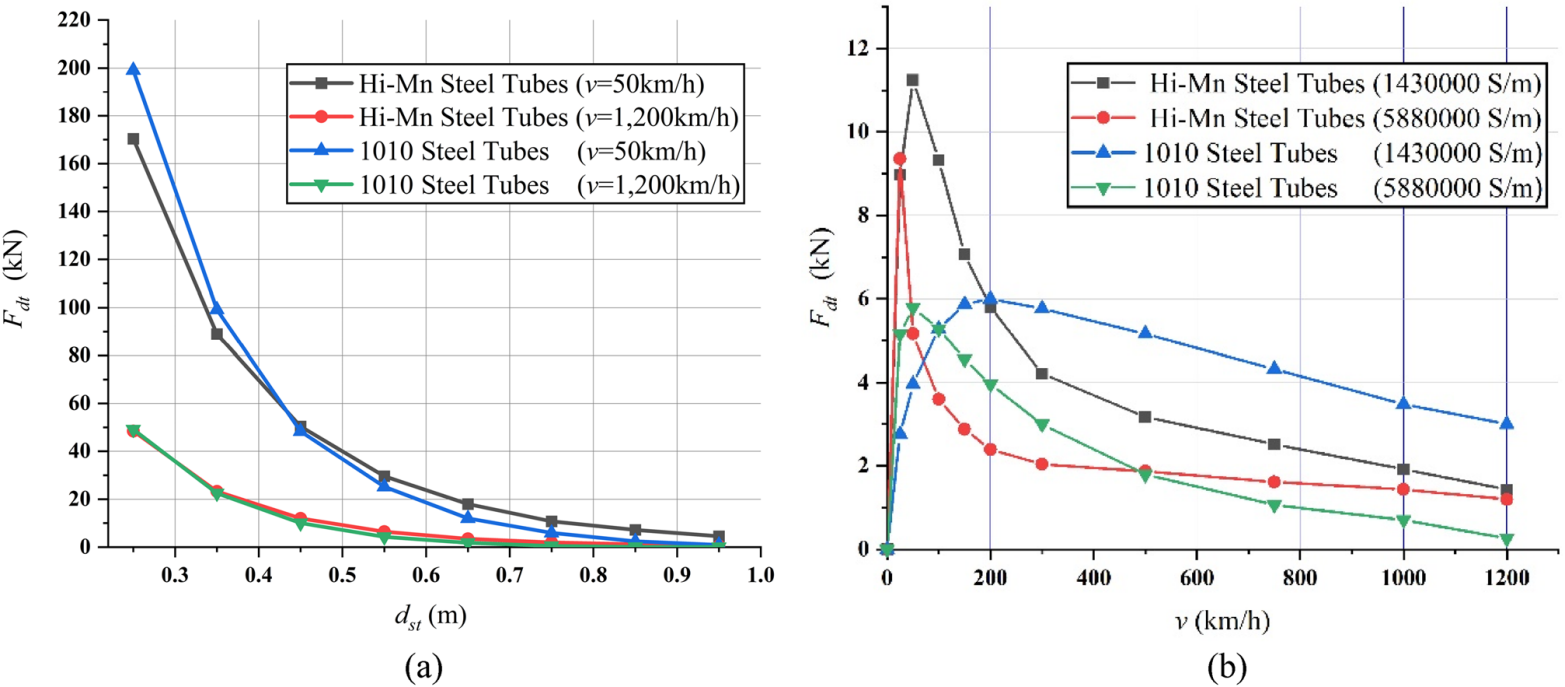
3D FEA simulation results of the EDFs produced by steel tubes, *F*_*dt*_, for full-scale Hyperloop including a 12 pole–6 module HTS magnet. (**a**) *F*_*dt*_ produced by Hi-Mn and AISI 1010 steel tubes corresponding to various *d*_*st*_ values at velocities of 50 and 1200 km/h, respectively. (**b**) *F*_*dt*_ produced by Hi-Mn, AISI 1010, and imaginary steel tubes corresponding to various operating velocities at *d*_*st*_ of 0.75 m.

The FEA simulations of *F*_*dt*_ produced by the Hi-Mn and AISI 1010 steel tubes were conducted at a velocity of 50 km/h, presenting the maximum *F*_*dt*_, and at a velocity of 1200 km/h presenting the minimum *F*_*dt*_ within the operational velocities, as shown in Fig. 19a. Specifically, the *F*_*dt*_ produced by the Hi-Mn steel tubes is higher than that of the AISI 1010 steel tubes from the *d*_*st*_ from 0.45 to 0.95 m while the *F*_*dt*_ produced by the AISI 1010 steel tubes is higher than that of the Hi-Mn steel tubes before the *d*_*st*_ of 0.45 m at the *v* of 50 km/h. At the *v* of 1200 km/h, the *F*_*dt*_ produced by the Hi-Mn steel tubes is higher than that of the AISI 1010 steel tubes with the *d*_*st*_ from 0.35 to 0.95 m.

**Figure 20:**
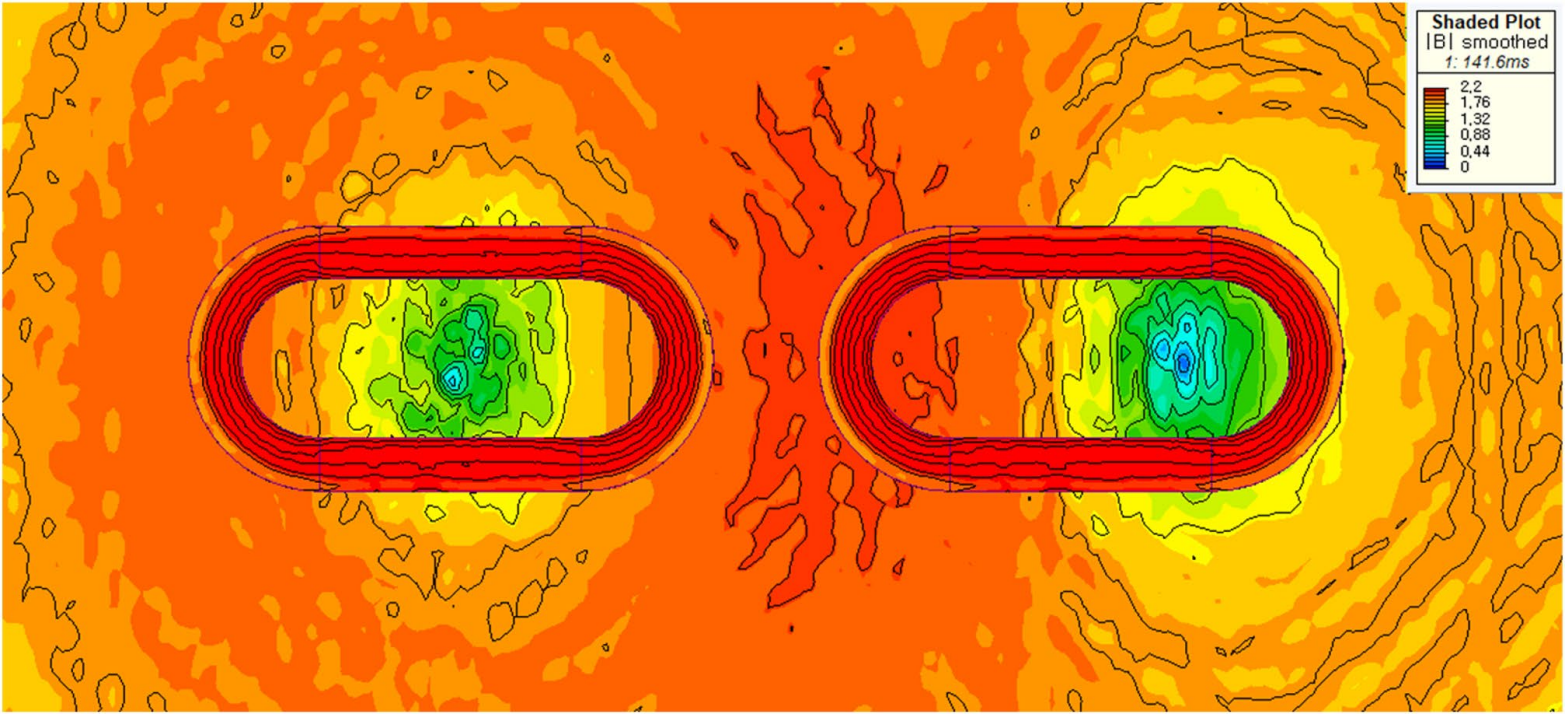
3D FEA simulation result of the 3D half-model for shaded *B* plot of the AISI 1010 steel tube using a 2 pole-1 module HTS magnet with *v* of 50 km/h at *d*_*st*_ of 0.75 m.

As shown in Fig. 19b, the simulations of *F*_*dt*_ produced by the Hi-Mn and AISI 1010 steel tubes were performed at the *d*_*st*_ of 0.75 m within the operational velocities. Generally, the *F*_*dt*_ produced by the Hi-Mn steel tubes is higher than that produced by the AISI 1010 steel tubes, and the maximum *F*_*dt*_ produced by the Hi-Mn steel tubes is approximately two times higher than that produced by the AISI 1010 steel tubes at the velocity of 50 km/h. The AC resistance of the AISI 1010 steel tubes is higher than that of the Hi-Mn steel tubes, as shown in Fig. 7, even though the hysteresis loss^36,37^ exists on the AISI 1010 steel tubes. This is because the AISI 1010 steel tubes are not fully saturated as shown in Fig. 20. In detail, for given induced voltages on the AISI 1010 steel tubes generated by moving HTS magnets, the high AC resistance of the AISI 1010 steel tubes results in the lower induced eddy currents, which cause the lower *F*_*dt*_ than that of the Hi-Mn steel tubes.

Moreover, the simulations of *F*_*dt*_ produced by the imaginary materials, i.e., Hi-Mn steel tubes having conductivity of 5,880,000 S/m and AISI 1010 steel tubes having conductivity of 1,430,000 S/m, were conducted at the *d*_*st*_ of 0.75 m within the operational velocities to determine potential materials for the vacuum tubes.

The results clarify that the higher conductivity for the same material, the faster decrease in the *F*_*dt*_. Also, the *F*_*dt*_ produced by the Hi-Mn steel tubes with the conductivity of 5,880,000 S/m could be one of the imaginary materials to be developed by steelmaking companies due to a sharp decline in the *F*_*dt*_ after the maximum *F*_*dt*_ at the velocity of 25 km/h.

In conclusion, when *d*_*st*_ is approximately 0.75 m, the maximum *F*_*dt*_ of 6 kN, produced by the AISI 1010 steel tubes with the conductivity of 5,880,000 S/m, which are already in mass production, could be acceptable because the maximum *F*_*dt*_ is under the allowable drag force, i.e., 25% of the designed propulsion force of 40 kN.

### Electromagnetic drag forces by steel tubes and EDS rails

**Figure 21:**
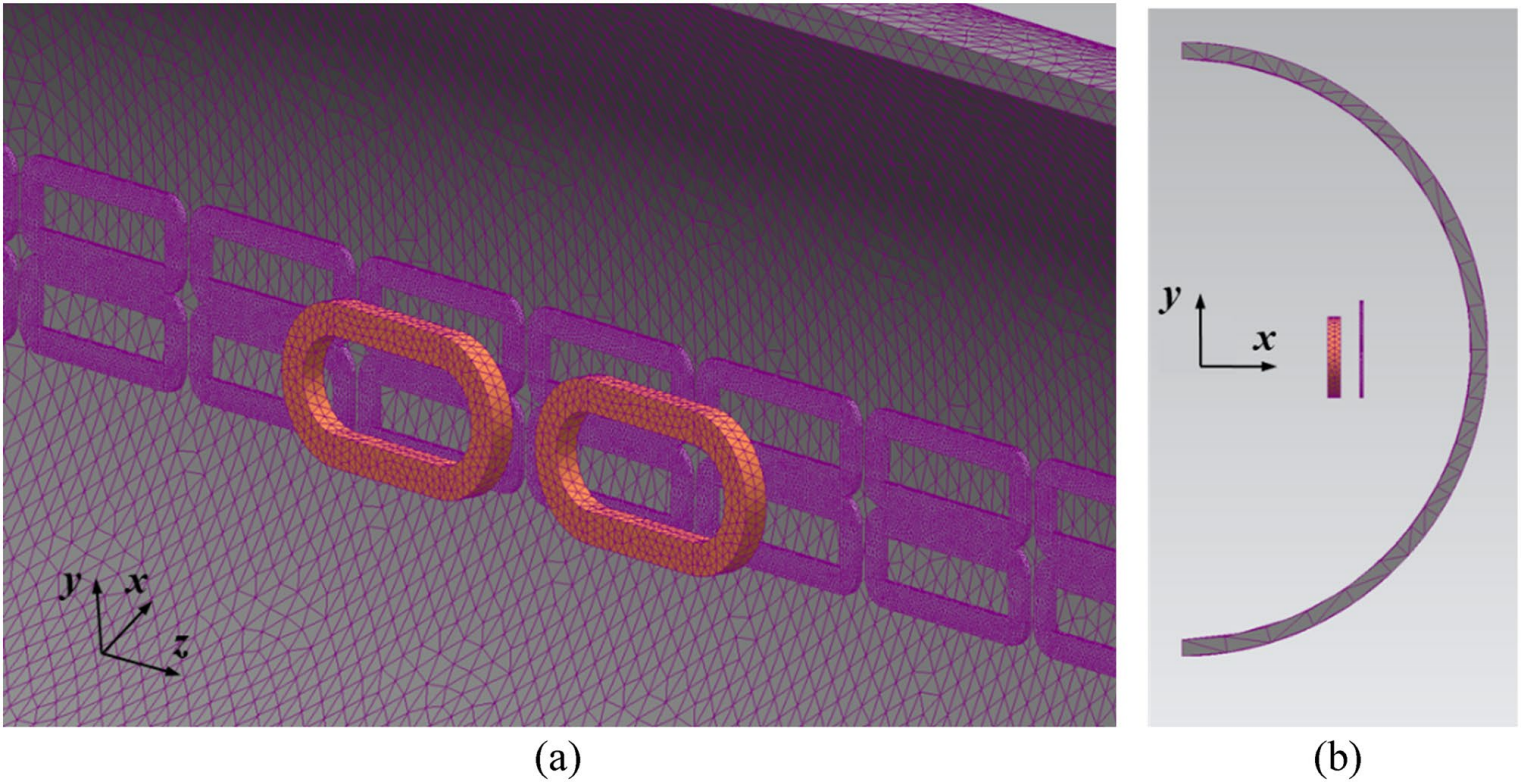
3D half-models (Siemens MagNet) of EDFs produced by EDS rails, *F*_*dl*_, and steel tubes, *F*_*dt*_, with a simplified 2 pole–1 module HTS magnet. (**a**) Bird’s eye view. (**b**) Front view.

This section presents an analysis of the total EDFs, *F*_*d*_, exerted by steel tubes and EDS rails with the AISI 1010 steel and Hi-Mn steel tubes along with its operating velocities. The 3D half-model including a 2 pole–1 module HTS magnet with steel tubes and EDS rails is adopted, similar to the previous simulation model of rebars, as shown in Fig. 21.

**Figure 22:**
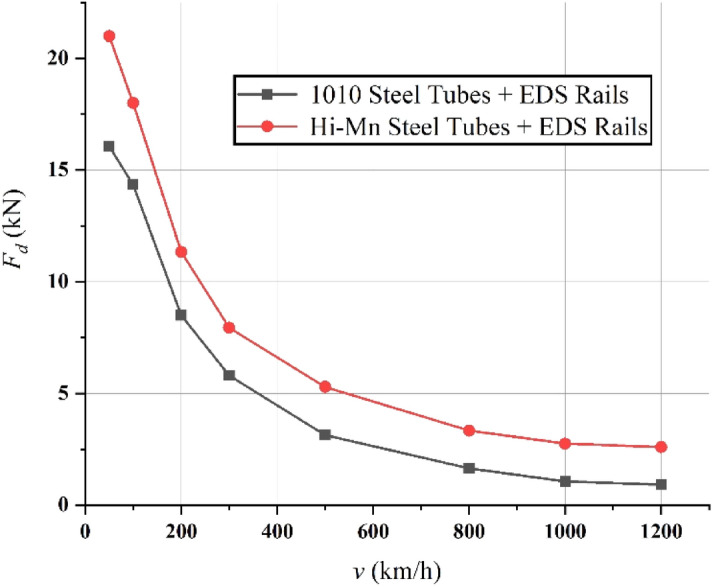
3D FEA simulation results of *F*_*d*_ produced by tubes as well as EDS rails for full-scale Hyperloop including a 12 pole–6 module HTS magnet along to various operating velocities at *d*_*st*_ of 0.75 m and Δ*z* of 0.05 m.

FEA simulations of the *F*_*d*_ produced by the Hi-Mn steel and AISI 1010 steel tubes were conducted along with those of the EDS rails at *d*_*st*_ of 0.75 m and Δ*z* of 0.05 m, as shown in Fig. 22. In line with its operating velocities, the *F*_*d*_ produced by the Hi-Mn steel tubes with EDS rails is generally higher than that produced by the AISI 1010 steel tubes with EDS rails. In general, the *F*_*d*_ produced by the EDS rails are evenly added for both of the AISI 1010 and Hi-Mn steel tubes, therefore, the results mainly come from the low AC resistance of the Hi-Mn steel tubes due to its skin effect, as mentioned in Section “Electromagnetic drag forces by steel tubes”. Before *v*_*l*_, the *F*_*d*_ produced by the Hi-Mn steel and AISI 1010 steel tubes with the EDS rails accounts for more than 25% of the designed propulsion forces of 40 kN, as shown in Fig. 22. However, the pods move on their own mechanical wheels with an offset Δ*z* of zero before a *v*_*l*_ of 150 km/h, which produces a *F*_*dl*_ of zero. Therefore, *F*_*f*_ and *F*_*dt*_ are only applied below *v*_*l*_; conversely, both *F*_*dl*_ and *F*_*dt*_ are applied after the pods levitate with a Δ*z* of 0.05 m.

From 0 to 150 km/h, the total drag forces, *F*_*td*_, contain aerodynamic drag forces, *F*_*a*_, mechanical friction forces, *F*_*f*_, and electromagnetic drag forces originating from rebars, *F*_*dg*_, and steel tubes, *F*_*dt*_. Before *v*_*l*_, the *F*_*td*_ can be simplified as follows:

20-1 $$ F_{td} = F_{a} + F_{f} + F_{dg} + F_{dt} \cong F_{f} + F_{dt} . $$

From 150 to 1200 km/h, *F*_*td*_ include *F*_*a*_, *F*_*dg*_, *F*_*dt*_ and electromagnetic forces originating from EDS coils, *F*_*dl*_. After *v*_*l*_, *F*_*td*_ can be summarized as follows:

20-2 $$ F_{td} = F_{a} + F_{dg} + F_{dt} + F_{dl} \cong F_{dt} + F_{dl} . $$

**Figure 23:**
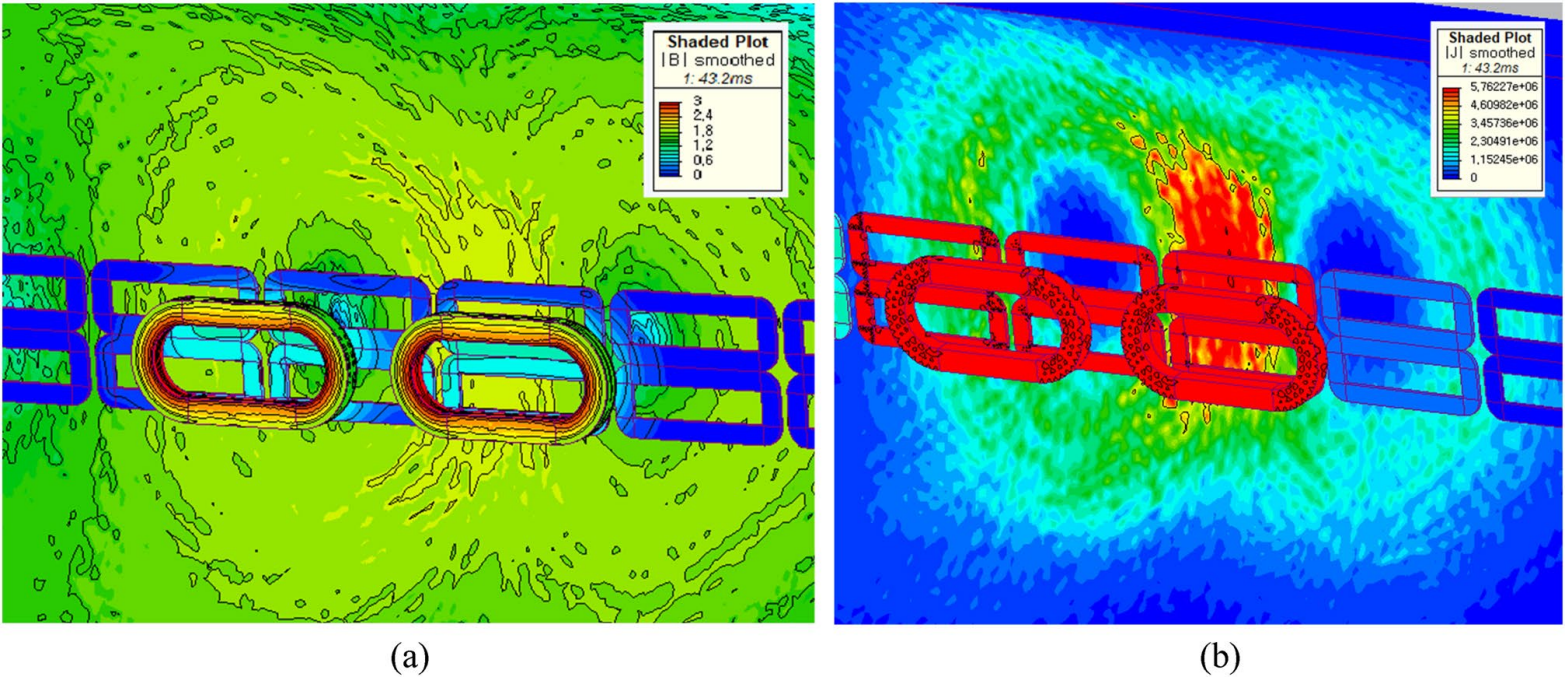
3D FEA simulation results of the 3D half-models (Siemens MagNet) with a 2 pole-1 module HTS magnet with *v* of 200 km/h at *d*_*st*_ of 0.75 m. (**a**) Shaded *B* plot and (**b**) shaded *J* plot for the AISI 1010 steel tube with EDS rails.

In summary, after the pods levitate with a Δ*z* of 0.05 m, the maximum *F*_*d*_ of 8.2 kN produced by the AISI 1010 steel tubes with the EDS rails could be below the acceptable drag force of 10 kN, which is 25% of the designed propulsion force of 40 kN. As shown in Fig. 23, the shaded *B* and *J* plots are presented at the maximum *F*_*d*_ of 8.2 kN produced by the AISI 1010 steel tubes with the EDS rails.

At the same time, the Hi-Mn steel tubes with EDS rails could be one of choices because there are no attraction forces between HTS magnets and tubes, which are the main factors to degrade the dynamic stability of pods; however, for the commercialization of Hyperloop, the AISI 1010 steel tubes would be better choice for now considering the fact that they are two times more cost-effective than the Hi-Mn steel tubes.

**Figure 24:**
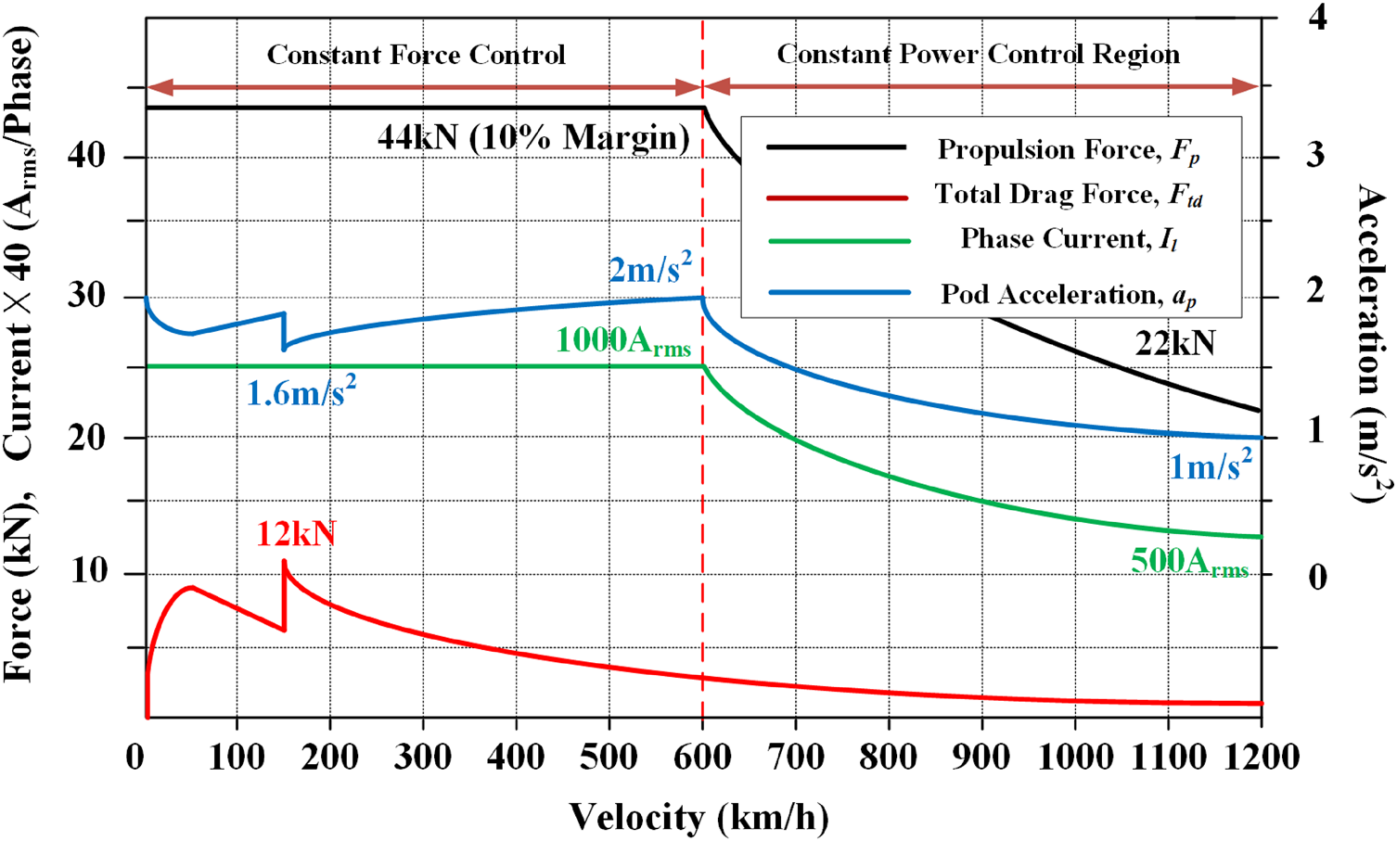
Profile of *F*_*p*_, *F*_*td*_, *I*_*l*_, and *a*_*p*_ for the proposed full-scale Hyperloop with the Hi-Mn rebars, AISI 1010 steel tubes and EDS rails.

As shown in Fig. 24, to reach the maximum velocity in a short time, *a*_*p*_ should be sufficiently high enough; simultaneously, *a*_*p*_ should be below the limitation of 0.2 G. Otherwise, it is likely to degrade the riding-comfort and make passengers feel uncomfortable when it lasts for several minutes. Moreover, for the efficient usage of the power supply systems, the two control schemes are used, i.e., constant force control and constant power control, which are divided by *v* of 600 km/h. Using the constant force control, the constant *I*_*l*_ of 1000 *A*_*rms*_ flows into LSM ground rails to generate the constant *F*_*p*_ forces of 44 kN on HTS magnets from 0 to 600 km/h. Additionally, with an increase in velocity from 600 to 1200 km/h, *I*_*l*_ gradually decreases to maintain the maximum capacity of inverters^18^.

## Conclusions

This paper presented a comprehensive analysis of the EDFs generated by HTS magnets, which account for most of the drag forces on the pods of Hyperloop. The analysis results of the total drag force apart from the EDF, that is, the mechanical and aerodynamic drag forces, indicated that after *v*_*l*_*,* the mechanical and aerodynamic drag forces could be negligible below 1.2 kN with an optimum BR of 0.6 at a vacuum tube pressure of 0.001 atm. Three different models with rebars, steel tubes, and EDS rails were constructed, and 3D FEA simulations (Siemens MagNet) were performed to calculate each EDF to obtain the total EDF. By adopting insulated Hi-Mn steel rebars, the attraction forces between the HTS magnets and rebars, which degrade the dynamic stability of pods, could be reduced to zero at zero velocity, and the EDF of 0.5 kN produced by the Hi-Mn steel rebars was negligible within its various operating velocities. Additionally, when the distance between the HTS magnets and steel tubes was approximately 0.75 m, the maximum EDF of 6 kN produced by the AISI 1010 steel tubes was obtained. The maximum EDF of 8.2 kN produced by the AISI 1010 steel tubes with EDS rails could be below the acceptable drag force of 10 kN, which is 25% of the designed propulsion force of 40 kN. Therefore, the 3D FEA simulation results of the total magnetic drag forces presented considerable insights for the general analysis and design of rebars, steel tubes, and EDS rails to minimize the total electromagnetic forces for Hyperloop using HTS magnets, which can contribute significantly to the commercialization of Hyperloop.

When *v* increases, *F*_*ag*_ is inversely proportional to the square of the operating velocity, whereas *F*_*al*_ is directly proportional to the square of the operating velocity in steel tubes; this effect is strongly related to the dynamics of pods. After the optimal design for the guidance and levitation forces, the total drag forces on the HTS magnets made by all the magnetic couplings between the rebars, steel tubes, and EDS rails should be considered. Moreover, electromagnetic shield in the passenger cabin should be also considered for the commercialization of Hyperloop. These topics will be discussed in future studies.

## Data Availability

The datasets generated from this work are available from the corresponding author on reasonable request.

## Abbreviations

ADF: Aerodynamic drag forces
BR: Blockage ratio
CFD: Computational fluid dynamics
EDF: Electromagnetic drag force
EDS: Electrodynamic suspension
EMS: Electromagnetic suspension
EPLS: Electromagnetic propulsion and levitation system
FRP: Fiber-reinforced plastic
Hi-Mn: High-manganese
HTS: High-temperature superconducting
HVAC: Heating, ventilation and air conditioning
KRRI: Korea Railroad Research Institute
LSM: Linear synchronous motors
LIM: Linear induction motors
MFF: Mechanical friction forces
